# Integrated WGCNA, Network Pharmacology, and UPLC-MS/MS Profiling for Investigating the Antitumor Effects of the Kansui Radix Dichloromethane Fraction Against Renal Cell Carcinoma with Experimental Validation

**DOI:** 10.3390/ijms27167325

**Published:** 2026-08-16

**Authors:** Zhuoyang Cheng, Xinyue Chen, Shiqi Wang, Yunuan Bai, Jiangtao Zhou

**Affiliations:** 1 School of Pharmacy, Shanxi Medical University, Taiyuan 030001, China; 2 Medicinal Basic Research Innovation Center of Chronic Kidney Disease, Ministry of Education, Shanxi Medical University, Taiyuan 030001, China; 3 Shanxi Academy of Advanced Research and Innovation, Taiyuan 030032, China

**Keywords:** *Euphorbia kansui*, renal cell carcinoma, HIF/VEGF signaling pathway, WGCNA

## Abstract

Kansui Radix, the root of *Euphorbia kansui*, was first described in Shen Nong Ben Cao Jing as a traditional Chinese medicine, known for its effects of expelling water, reducing edema, and dissipating masses. It is traditionally indicated for conditions such as “watery accumulation” and “abdominal masses (zheng-jia)”, which share certain similarities with the clinical manifestations of renal cell carcinoma (RCC), including renal masses, edema, and body cavity effusion. Despite the recognized antitumor effects of Kansui Radix, the pharmacological basis and specific molecular mechanisms underlying its inhibition of RCC progression remain unclear. The goal of this study was therefore to evaluate the antitumor efficacy of Kansui-DCM in RCC and to explore its potential mechanism. To this end, the chemical composition of the dichloromethane fraction of Kansui Radix (Kansui-DCM) was characterized by UPLC-MS. The antiproliferative effects of Kansui-DCM on 786-O and RENCA cells were evaluated using the CCK-8 assay. Apoptotic morphology, apoptosis rate, cell migration and invasion abilities were assessed. An RCC mouse model was established, and tumor growth and histopathological staining were evaluated after drug administration. Immunohistochemistry, transcriptomic analysis, WGCNA (weighted gene co-expression network analysis), network pharmacology, and immunofluorescence were employed to investigate the molecular pathway. Protein and gene expression were analyzed by Western blot and qRT-PCR, respectively. Finally, the interactions between the active components and key targets were substantiated through molecular docking and molecular dynamics simulations. A total of 1397 compounds were detected in Kansui-DCM by UPLC-MS. In vitro experiments demonstrated that Kansui-DCM inhibited the proliferation of 786-O and RENCA cells in a dose-dependent manner, induced apoptosis, and suppressed cell invasion and migration in RENCA cells. Treatment with Kansui-DCM markedly suppressed tumor growth in vivo, as reflected by a reduction in tumor volume. Immunohistochemical analysis indicated that the expression levels of CD31, COX-2, and Ki67 were markedly decreased, while the expression level of CD8 was significantly increased. Integrated analysis using WGCNA and network pharmacology predicted that Kansui-DCM may exert its effects through the regulation of the HIF/VEGF signaling pathway, which was further validated by immunofluorescence, Western blot, and qRT-PCR assays. Molecular docking and molecular dynamics simulations demonstrated stable interactions between multiple active components of Kansui-DCM and key targets such as VEGFR2 and HIF-2α. In conclusion, these findings suggested that Kansui-DCM exerts anti-RCC effects associated with regulation of the HIF/VEGF pathway.

## 1. Introduction

Renal cell carcinoma (RCC), the predominant form of kidney cancer, ranks as the 12th most common malignancy worldwide, with approximately 400,000 new cases and 175,000 deaths occurring annually [1]. RCC is a heterogeneous malignancy that encompasses multiple subtypes; among them, clear cell RCC (ccRCC) is the most prevalent and constitutes approximately 70–75% of cases [2]. While localized RCC is often curable through surgical resection, about 30% of patients develop metastatic disease, which historically carries a poor prognosis [3]. The modern clinical landscape has been revolutionized by the understanding of key genetic drivers such as VHL inactivation in ccRCC [4]. This has led to a “golden age” of treatment, evolving from targeted therapies against VEGF and mTOR to the recent integration of immune checkpoint inhibitors [2]. Current first-line standards for advanced disease frequently involve potent immunotherapy and tyrosine kinase inhibitor combinations, such as pembrolizumab plus axitinib, which have significantly improved patient outcomes [5]. However, primary and acquired resistance to existing therapies remains a major obstacle, which leads to disease progression for many patients [6]. Therefore, more effective drugs to treat RCC are urgently needed.

Traditional Chinese medicines offer significant anticancer potential through diverse bioactive compounds that target multiple dysregulated pathways [7,8]. The dried root of *Euphorbia kansui*, known as Kansui Radix, has been used in traditional Chinese medicine for over 2000 years [9] to expel fluid retention [10]. Phytochemical studies have led to the isolation and identification of over 100 active constituents from Kansui Radix, including diterpenoids, triterpenes, and phenolic derivatives [11]. Modern studies have revealed that Kansui Radix has various biological activities, such as antitumor, anti-epilepsy, anti-viral, anti-inflammatory activity [12]. Among them, the antitumor activity has attracted considerable attention because of the potential activity against a wide range of tumors [13,14,15]. Compounds from Kansui Radix can trigger cell death in HepG2 cells and suppress both M1 and M2 macrophage polarization [16] and exhibit potent antiproliferative activity against various cancer cells, including HCT-116, MKN-45 [16], MCF-7, and DU145 [17]. However, the involvement of Kansui Radix in RCC is still unknown. Notably, RCC is typically driven by the constitutive activation of the hypoxia-inducible factor (HIF) and vascular endothelial growth factor (VEGF) signaling axis, a well-established hallmark of ccRCC pathogenesis. This axis constitutes the central molecular machinery that governs cellular oxygen sensing and regulates angiogenesis [18].

Under hypoxic conditions, the α subunit of the heterodimeric transcription factor *HIF* (*HIF-1α* or *HIF-2α*) becomes stable and activates downstream genes, with *VEGF* being a critical target [19,20]. In many solid tumors, constitutive activation of the HIF-VEGF pathway, often driven by genetic alterations such as *VHL loss*, promotes robust angiogenesis, supplying oxygen and nutrients to support uncontrolled tumor growth [21,22]. In clear cell RCC, *VHL* inactivation is a hallmark event that leads to persistent HIF stabilization and VEGF overexpression, making this pathway a key therapeutic target. Moreover, HIF-driven VEGF signaling not only facilitates neovascularization but also metabolic reprogramming, immune evasion, and increased invasiveness and metastatic potential in cancer cells [18]. Consequently, hyperactivation of this pathway often drives tumor aggressiveness, portends a poor prognosis, and confers resistance to conventional therapies [23].

Considering the pivotal involvement of HIF/VEGF signaling in the development of RCC, together with our initial network pharmacology findings, we hypothesized that Kansui Radix might exert anti-RCC effects through this pathway. To test this, we systematically analyzed the chemical composition of the active dichloromethane fraction of Kansui Radix (Kansui-DCM) using UPLC-MS. A network pharmacology model based on weighted gene co-expression network analysis (WGCNA) was constructed to predict potential mechanisms. For in vitro studies, we employed 786-O cells (a *VHL*-mutant human ccRCC line) to assess tumor cell-intrinsic responses; for in vivo studies, we used the RENCA syngeneic model to evaluate antitumor efficacy in an immunocompetent setting, thereby allowing parallel examination of both direct and immune-mediated effects. The objective of this combined in vivo and in vitro study was to elucidate the anti-RCC mechanism of Kansui Radix and to provide mechanistic support for its clinical translation.

## 2. Results

### 2.1. UPLC-MS Analysis of Kansui-DCM

Screening of the cytotoxic activities of Kansui Radix fractions by CCK-8 assay revealed that Kansui-DCM was the principal active fraction (Figure S1, supporting data are provided in the Supplementary Materials) and it was therefore selected for further investigation.

**Figure 1:**
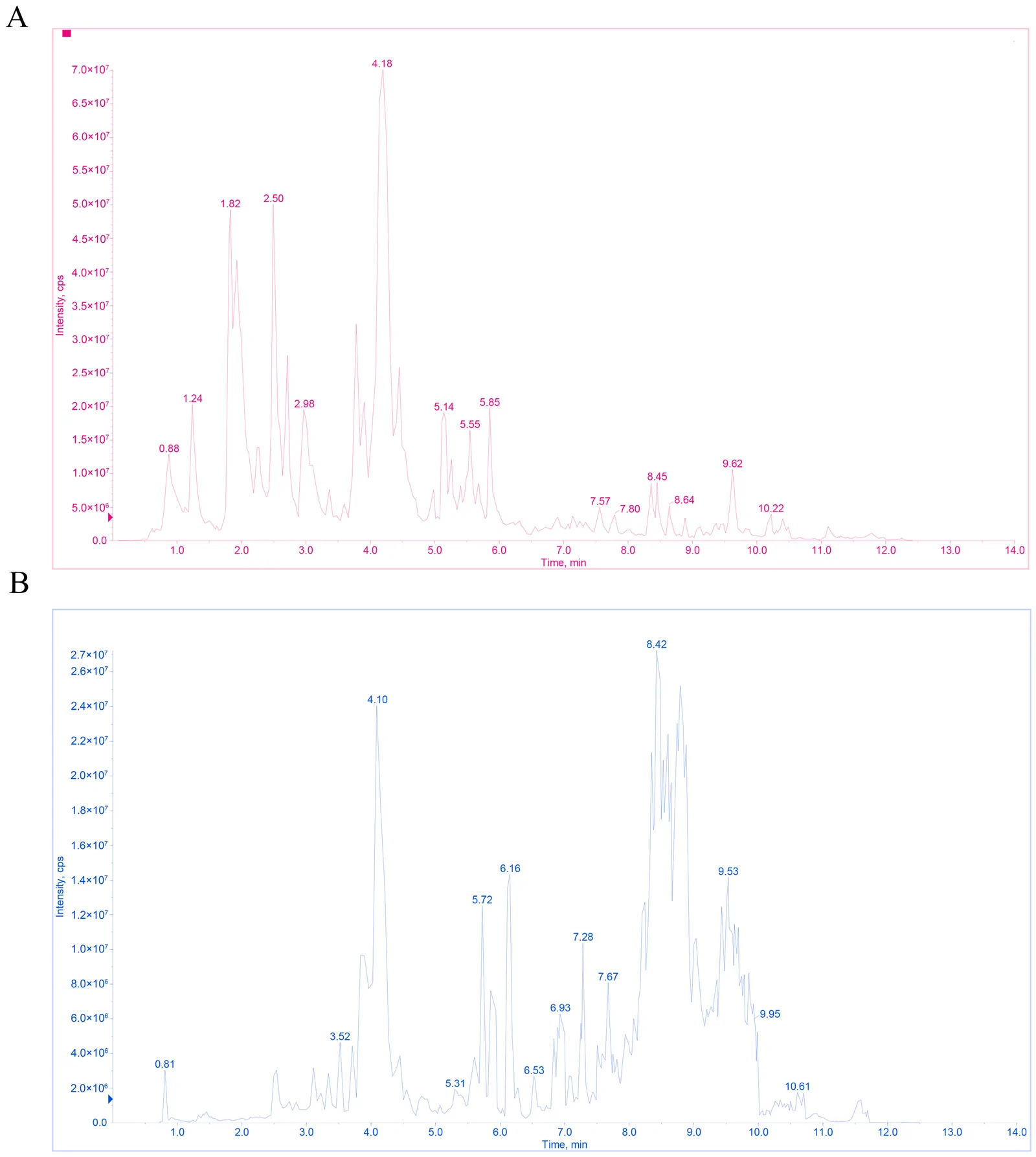
UPLC-MS spectra of Kansui-DCM. (**A**) Positive-ion mode; (**B**) negative-ion mode.

UPLC-MS analysis in both positive and negative modes resulted in the putative detection of 1397 compounds in Kansui-DCM (Figure 1), primarily comprising phenolic acids (235), flavonoids (243), quinones (22), lignans and coumarins (138), alkaloids (271), terpenoids (245), steroids (8), tannins (11), and others (224) under UPLC-MS detection. The top twenty compounds with a higher relative content based on peak area are listed in Table 1.

### 2.2. Kansui-DCM Induces Cell Apoptosis in 786-O and RENCA Cells

**Figure 2:**
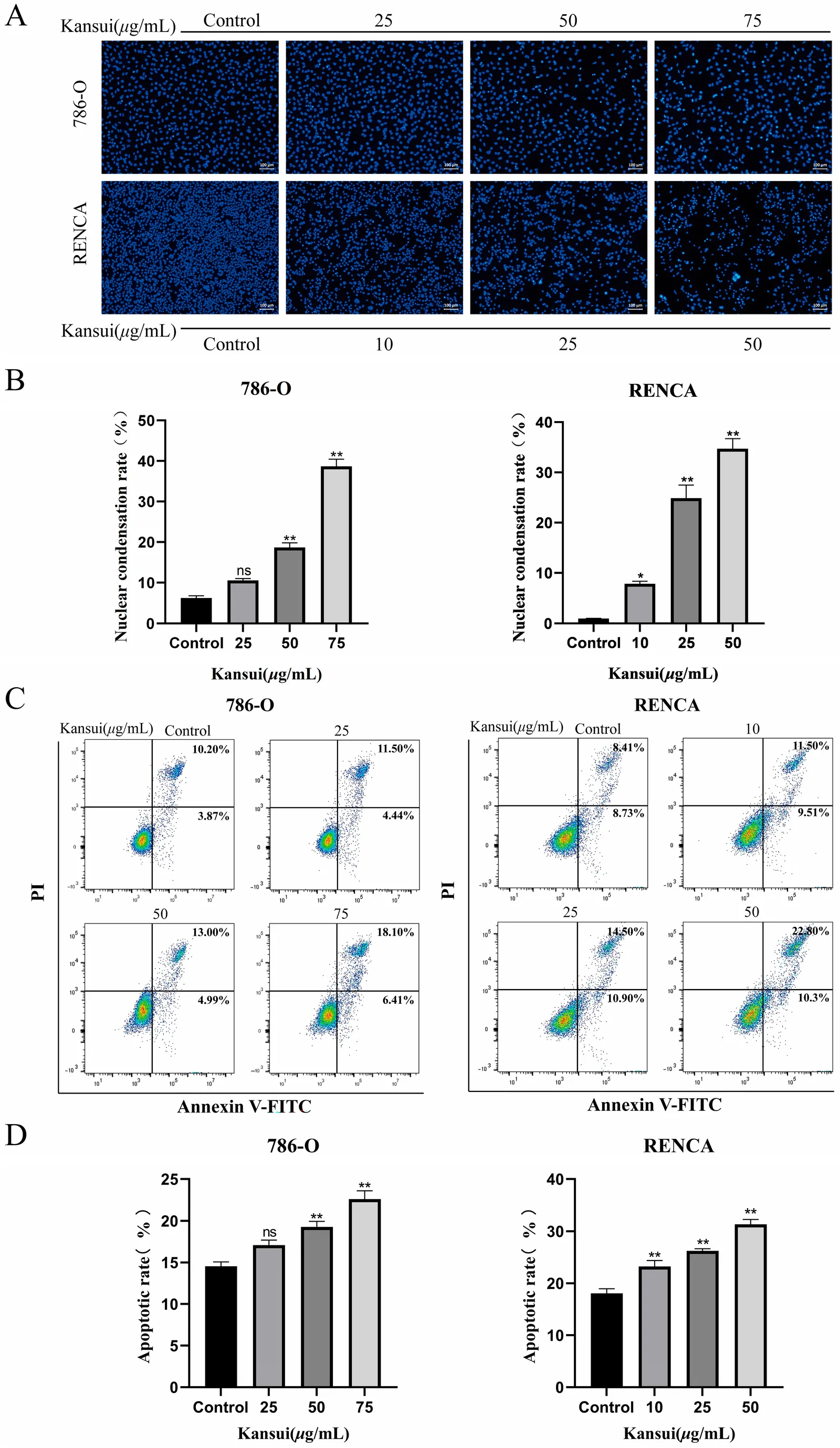
Effects of Kansui-DCM on the apoptosis of 786-O and RENCA cells; (**A**) Hoechst 33,258 staining detects apoptosis. The scale bar is 100 μm. Apoptotic cells exhibit bright-blue fluorescence. (**B**) Calculated nuclear condensation rates of Hoechst-stained cells. (**C**) Detection of apoptosis by Annexin V-FITC and PI double staining via flow cytometry. (**D**) Apoptotic cell percentages, measured by flow cytometry and averaged over three independent experiments (mean ± SD), were compared with the control group. Statistical significance was determined by one-way ANOVA with Dunnett’s post hoc test. * *p* < 0.05 and ** *p* < 0.01, “ns” means no significance.

Hoechst 33,258 staining was employed to assess Kansui-DCM-induced apoptosis and necrosis in 786-O and RENCA cells. As shown in Figure 2A, the treatment led to evident nuclear condensation, fragmentation, and cell shrinkage, together with a notable rise in dead cells compared with the control. The proportion of apoptotic cells, as determined by ImageJ (version 1.54r) analysis of the captured images, was significantly greater in the Kansui-DCM-treated group than in the control group (786-O:6.25 ± 0.92%; RENCA:0.98 ± 0.07%) (*p* < 0.01). The nuclear condensation rates for 786-O cells exposed to increasing concentrations of Kansui-DCM were 10.57 ± 0.79%, 18.67 ± 1.98%, and 38.68 ± 3.06%, respectively, while the corresponding rates for RENCA cells were 7.86 ± 0.86%, 24.89 ± 4.48%, and 34.71 ± 3.48% (Figure 2B).

Consistent with the Hoechst staining results, flow cytometric analysis of Annexin V-FITC/PI-stained cells corroborated that Kansui-DCM treatment significantly promoted apoptosis in both 786-O and RENCA cells (Figure 2C). The apoptotic rates in 786-O cells treated with increasing concentrations of Kansui-DCM were 17.10 ± 1.03%, 19.28 ± 1.16%, and 22.60 ± 1.72%, respectively, whereas the control group exhibited an apoptotic rate of 14.56 ± 0.88%. Similarly, in RENCA cells, the apoptotic rates following Kansui-DCM treatment were 23.24 ± 1.96%, 26.23 ± 0.74%, and 31.33 ± 1.66%, compared with 18.06 ± 1.52% in the control group (*p* < 0.01) (Figure 2D).

### 2.3. Kansui-DCM Inhibits Metastasis in RENCA Cells

**Figure 3:**
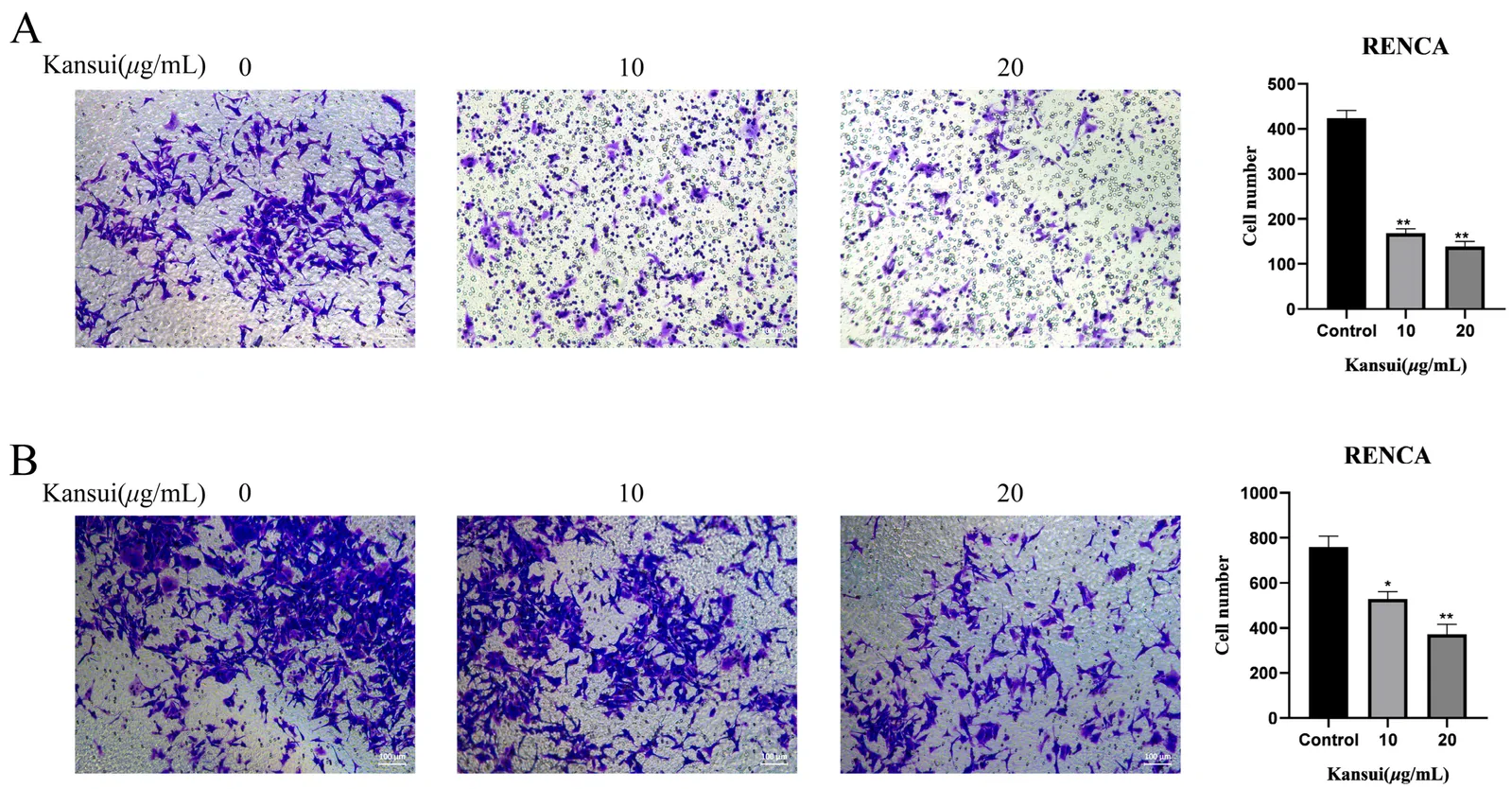
Effect of Kansui-DCM on migration and invasion of RENCA cells. (**A**) Representative images and quantification of cell migration. (**B**) Representative images and quantification of cell invasion. Cells were treated with indicated concentrations (10 and 20 μg/mL) for 24 h. Data were normalized to cell viability and presented as mean ± SD from three independent experiments. Statistical significance was determined by one-way ANOVA with Dunnett’s post hoc test. * *p* < 0.05, ** *p* < 0.01 vs. control group.

As shown in Figure 3, Kansui-DCM significantly inhibited both the migration and invasion of RENCA cells at 10 and 20 μg/mL in a dose-dependent manner (*p* < 0.05 or *p* < 0.01). The selected concentrations maintained cell viability at 90.7% and 79.7%, respectively, and all data were normalized to cell viability to exclude cytotoxic interference. In contrast, 786-O cells showed no significant inhibition of migration or invasion at non-cytotoxic concentrations after normalization.

### 2.4. Kansui-DCM Suppresses Tumor Growth in a RENCA Subcutaneous Xenograft Mouse Model

**Figure 4:**
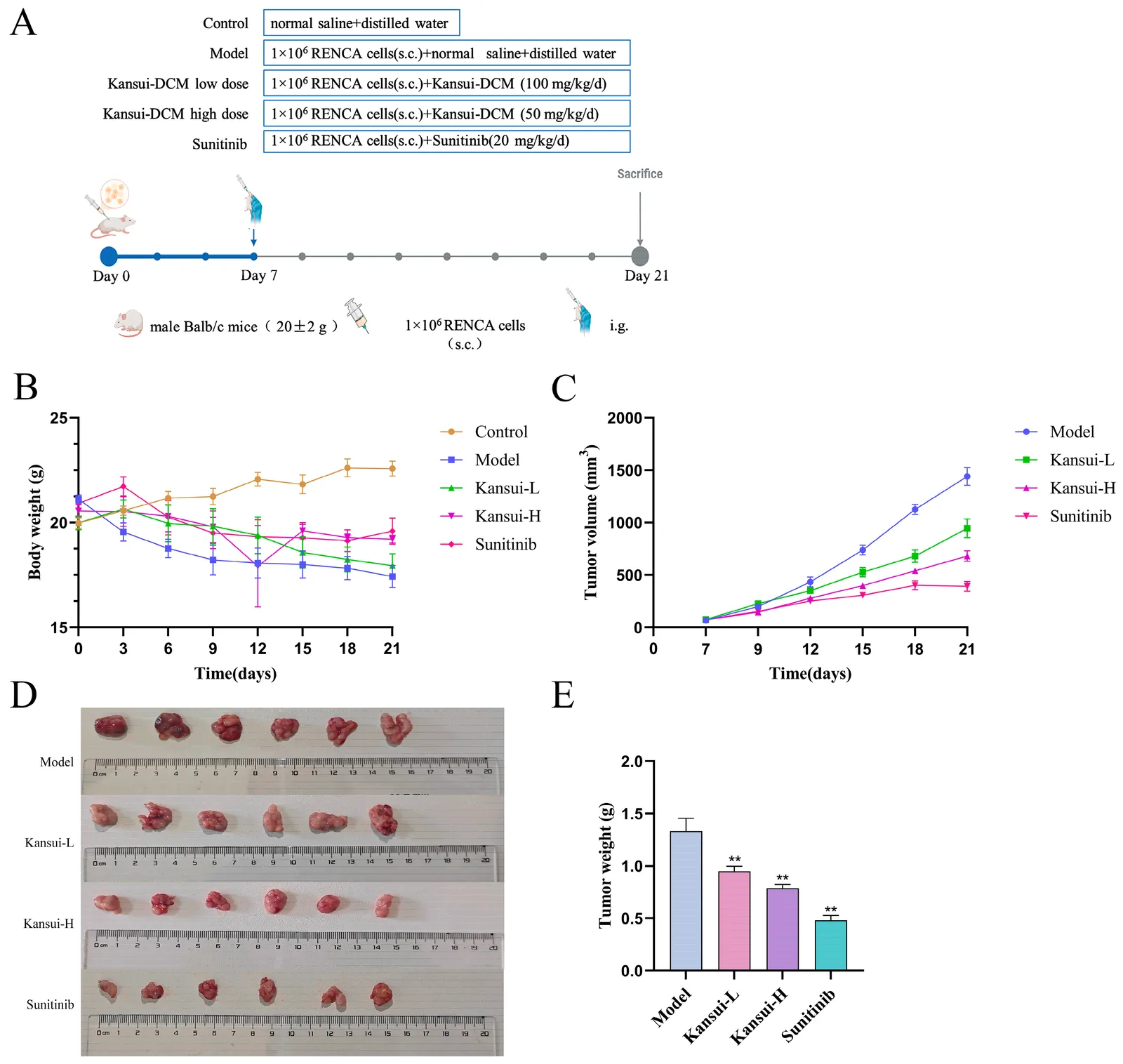
The in vivo tumor-inhibitory effect of Kansui-DCM. (**A**) Experimental design; (**B**) effect of Kansui-DCM on mouse body weight; (**C**) effect of Kansui-DCM on tumor volume in tumor-bearing mice; (**D**) representative images of xenograft tumors; (**E**) comparison of terminal tumor weights. Results are shown as mean ± SD of six replicates. Statistical significance was determined by one-way ANOVA with Dunnett’s post hoc test. ** *p* < 0.01 vs. the model group (*n* = 6).

Male Balb/c mice inoculated with RENCA cells served as the in vivo model to evaluate the antitumor activity of Kansui-DCM. The antitumor efficacy of Kansui-DCM was evaluated by monitoring body weight changes, as well as by measuring tumor volumes and tumor weights. Representative morphological changes in the tumors are depicted in Figure 4. No significant reduction in body weight was observed in mice receiving Kansui-L or sunitinib for 14 days, relative to the control group (Figure 4B). The tumor volumes in the model group, Kansui-L group, Kansui-H group, and sunitinib group were (1440.83 ± 210.09) mm^3^, (945.67 ± 216.00) mm^3^, (681.01 ± 124.32) mm^3^, and (392.27 ± 114.38) mm^3^, respectively (Figure 4C). Tumor volumes in the Kansui-L, Kansui-H, and sunitinib groups were markedly smaller than those in the model group (*p* < 0.01). As shown in Figure 4D,E, the mean tumor weight decreased significantly from 1.34 ± 0.29 g in the model group to 0.95 ± 0.12 g, 0.79 ± 0.09 g, and 0.48 ± 0.11 g in the Kansui-L, Kansui-H, and sunitinib groups, respectively.

### 2.5. Effects of Kansui-DCM on Histopathology and Tumor Marker Expression in the RENCA Subcutaneous Xenograft Model

**Figure 5:**
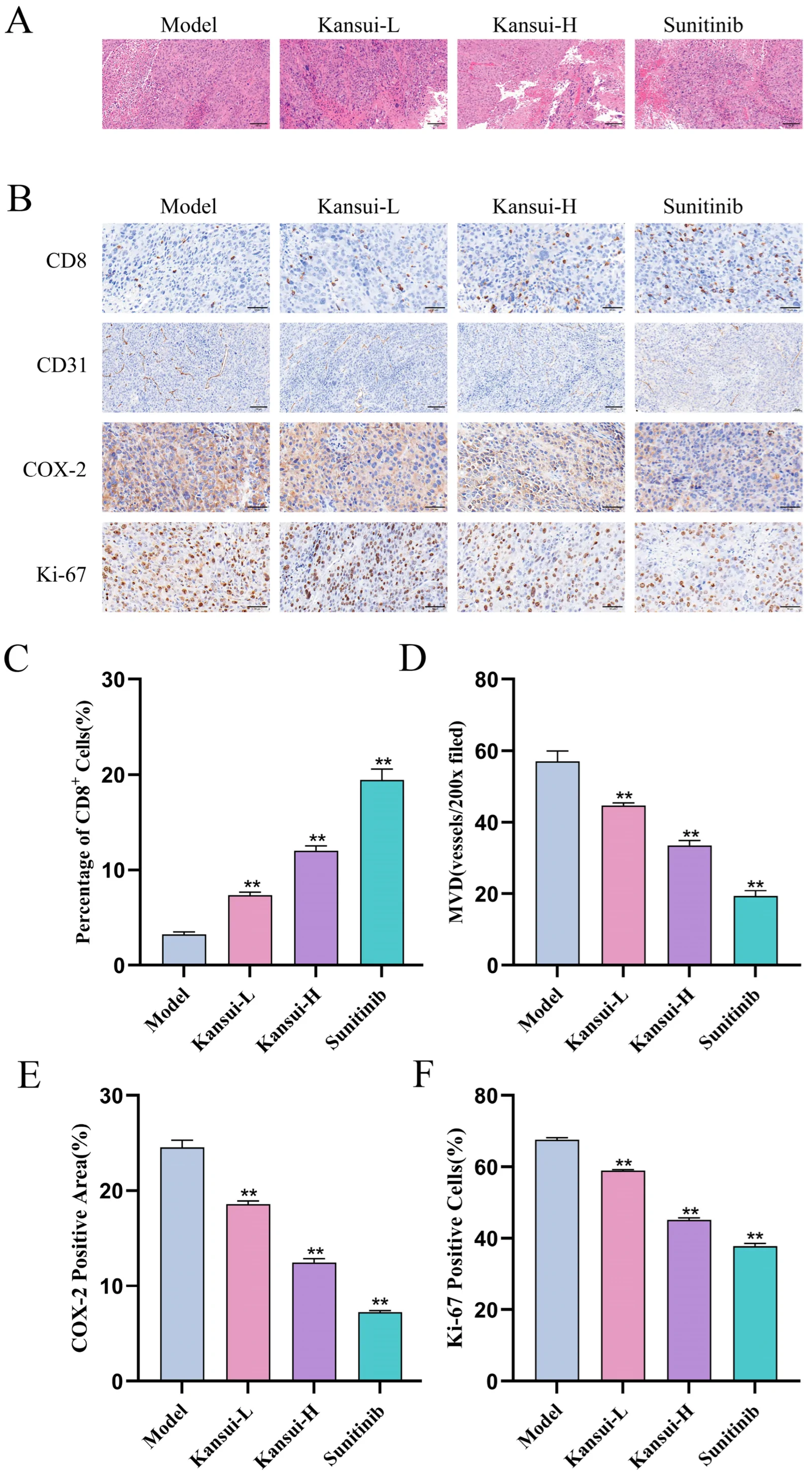
Effects of Kansui-DCM on histopathology and tumor marker expression in the RENCA subcutaneous xenograft model. (**A**) H&E pathology findings of tumor tissue from each group. Scale bars = 100 μm; (**B**) representative immunohistochemical staining of CD8, CD31, COX-2 and Ki-67 (magnification ×200); (**C**) percentage of CD8+ cells (%); (**D**) CD31 microvessel density; (**E**) COX-2 positive area (%); (**F**) Ki-67-positive cells (%). Results are shown as the mean ± SD of three parallel measurements for each group. Statistical significance was determined by one-way ANOVA with Dunnett’s post hoc test. ** *p* < 0.01 vs. the model group.

Hematoxylin and eosin (H&E) staining of tumor sections from the model group revealed that the tumor cells exhibited marked nuclear atypia, variation in cell size, prominent nucleoli, frequent mitotic figures, and neutrophilic infiltration (Figure 5A). These histological features are indicative of uncontrolled tumor proliferation and an adverse tumor microenvironment. On the contrary, the tumor tissues of mice in the Kansui-L group and Kansui-H group showed multifocal necrosis, decreased mitotic figures, and evidence of lymphocyte infiltration. The Sunitinib-treated group exhibited extensive necrosis and hemorrhage, consistent with its established anti-angiogenic mechanism of action. Collectively, these findings demonstrate that Kansui-DCM effectively suppresses the growth of renal cell carcinoma in vivo.

CD8, CD31, COX-2, and Ki-67 are established markers associated with tumor immune infiltration, angiogenesis, inflammation, and proliferation, respectively. Immunohistochemical analysis showed that Kansui-DCM significantly modified the expression of these markers in RENCA xenografts in a dose-dependent manner, as compared to untreated tumors (Figure 5B–F).

### 2.6. Integrative Transcriptomic and Network Pharmacological Analysis of Kansui-DCM in the RENCA Subcutaneous Xenograft Model

**Figure 6:**
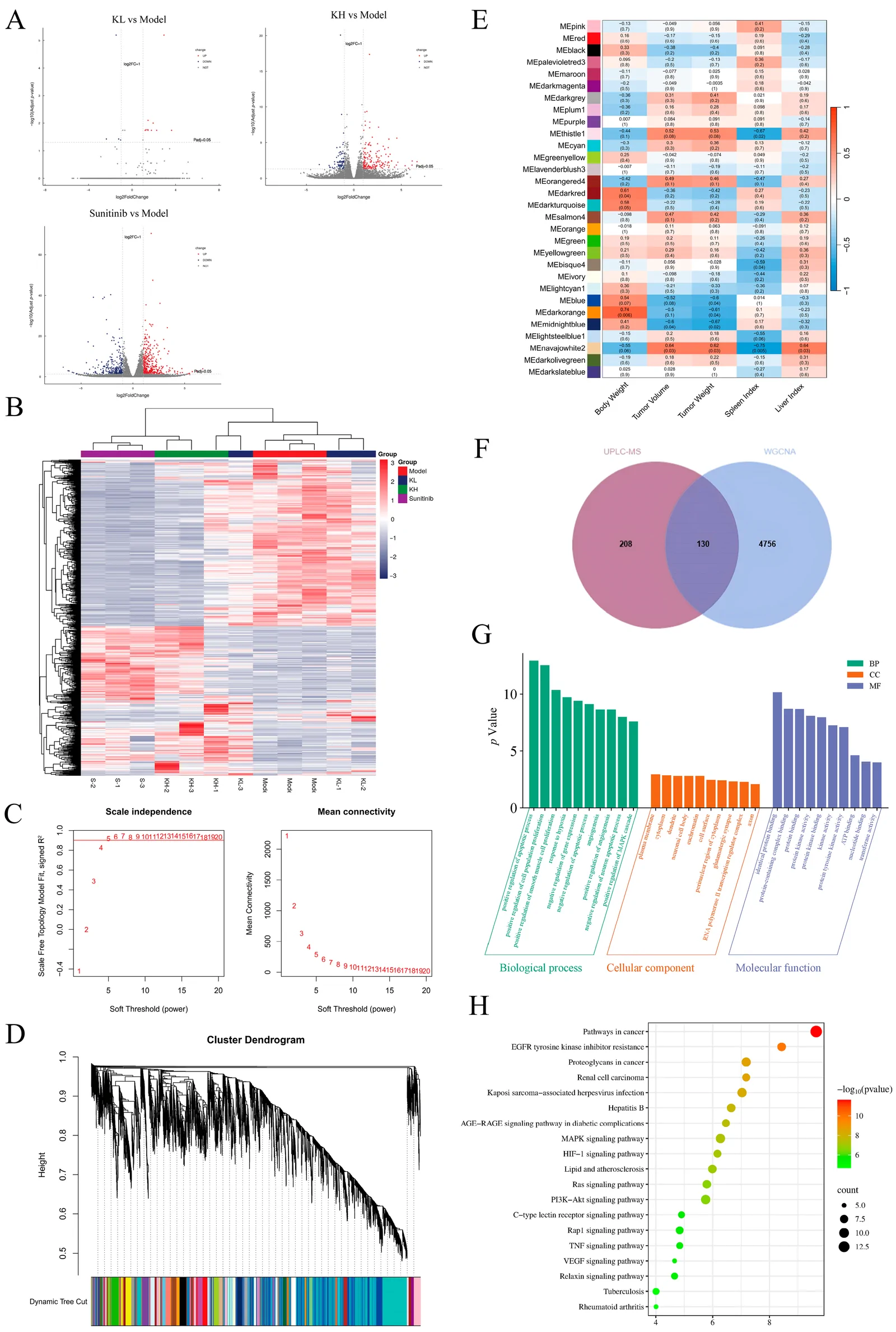
Transcriptomic profiling and network pharmacology analysis of Kansui-DCM in the RENCA subcutaneous xenograft model. (**A**) Volcano plot of differentially expressed genes: Kansui-L vs. model; Kansui-H vs. model; sunitinib vs. model; (**B**) clustering heatmap of differentially expressed genes; (**C**) R^2^ values and gene adjacency coefficients at different soft thresholds; (**D**) gene dendrogram constructed by hierarchical clustering based on gene adjacency coefficients; (**E**) common expression relationship between gene modules and various indicators; (**F**) Venn diagram; (**G**) GO analysis; (**H**) KEGG enrichment analysis.

Transcriptomic analysis via high-throughput sequencing was conducted to compare gene expression changes among tumor tissues from the model group, the two Kansui-DCM dose groups, and the sunitinib group. Hierarchical clustering analysis and the corresponding heatmap (Figure 6A,B) revealed distinct gene expression profiles among the experimental groups. Compared with the model group, 14 differentially expressed genes (DEGs) were identified in the low-dose Kansui-DCM group, of which 10 were upregulated and 4 were downregulated. In the high-dose Kansui-DCM group, 252 DEGs were detected, comprising 81 upregulated and 171 downregulated genes. The Sunitinib group exhibited 659 DEGs, with 413 genes upregulated and 246 downregulated.

A gene co-expression network was constructed by WGCNA based on transcriptomic data and five physiological metrics—body weight, tumor weight, tumor volume, spleen index, and kidney index—all obtained from the RENCA xenograft mouse model. According to the scale-free topology criterion and mean connectivity, the optimal soft-thresholding power was set to 5 (Figure 6C). The gene dendrogram, generated through hierarchical clustering of the adjacency matrix, is presented in Figure 6D. Dynamic tree cutting was subsequently applied to define gene modules. A total of 30 non-redundant co-expression modules, comprising 4886 genes, were obtained after merging similar modules (Figure 6E).

UPLC-MS analysis of Kansui-DCM identified a total of 1397 compounds. After database screening and deduplication, 75 candidate compounds were selected, corresponding to 338 potential target proteins. The intersection between these compound-related targets and the 4886 disease-associated genes identified through WGCNA was determined, yielding a set of 130 overlapping targets predicted to mediate the therapeutic effects of Kansui-DCM against renal cell carcinoma (Figure 6F).

As shown in Figure 6G, the potential targets were significantly enriched in biological processes such as positive regulation of the apoptotic process, cell population proliferation, and smooth muscle cell proliferation. In terms of cellular components, enrichment was observed in the plasma membrane, cytoplasm and dendrite. Molecular functions included identical protein binding, protein-containing-complex binding, and protein binding.

KEGG pathway enrichment analysis was subsequently performed on these intersecting targets, revealing 20 signaling pathways potentially associated with renal cell carcinoma (Figure 6H). Notably, the enriched pathways included the EGFR, PI3K-Akt, TNF, and VEGF signaling pathways. Given that VEGFA is a well-established downstream target of hypoxia-inducible factor (HIF) and plays a critical role in tumor angiogenesis [24], we further investigated the expression of core components within the HIF and VEGF signaling axes. Specifically, the protein and mRNA levels of HIF-1α, HIF-2α, VEGFA, and VEGFR2 were assessed in tumor tissues from VHL-wild-type mice using immunofluorescence staining, qRT-PCR, and Western blot analysis.

### 2.7. Immunofluorescence Analysis in RCC Mouse Tumor Tissue

**Figure 7:**
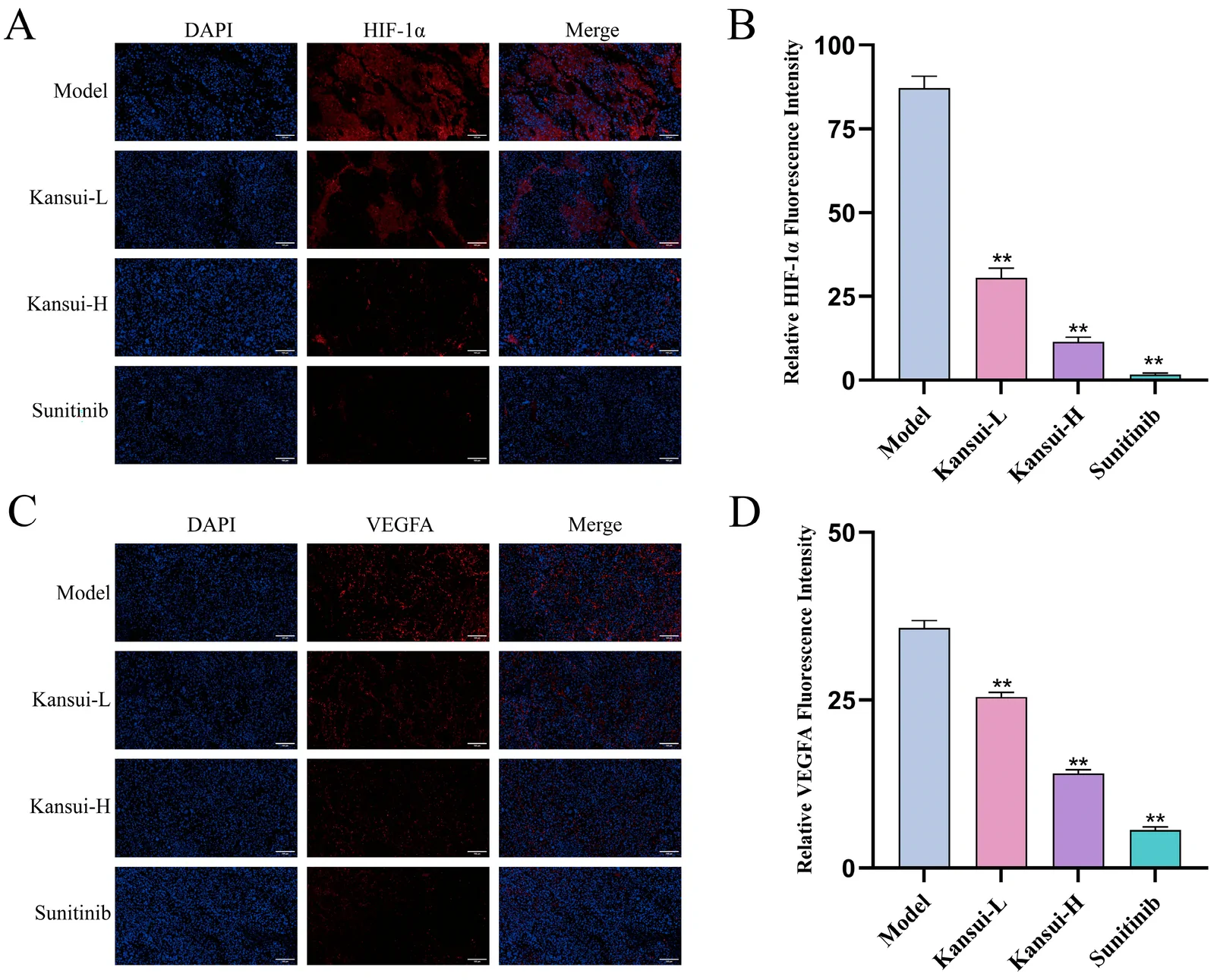
Effects of Kansui-DCM on HIF-1α and VEGFA in tumor-bearing mice. (**A**,**C**) Representative immunofluorescence staining of HIF-1α and VEGFA (magnification ×200); (**B**) relative HIF-1α fluorescence intensity; (**D**) relative VEGFA fluorescence intensity. The results are given as mean ± SD of triplicate assays for each group. Statistical significance was determined by one-way ANOVA with Dunnett’s post hoc test. ** *p* < 0.01 vs. the model group.

Immunofluorescence staining demonstrated that Kansui-DCM significantly downregulated both HIF-1α and VEGF-A in tumor tissues relative to the model group (*p* < 0.01) (Figure 7). Our data imply that Kansui-DCM may alleviate the hypoxic tumor microenvironment and thereby suppress tumor proliferation.

### 2.8. Kansui-DCM Inhibits the Activation of the HIF/VEGF Pathway in the RENCA Subcutaneous Tumor Xenograft Mouse Model

**Figure 8:**
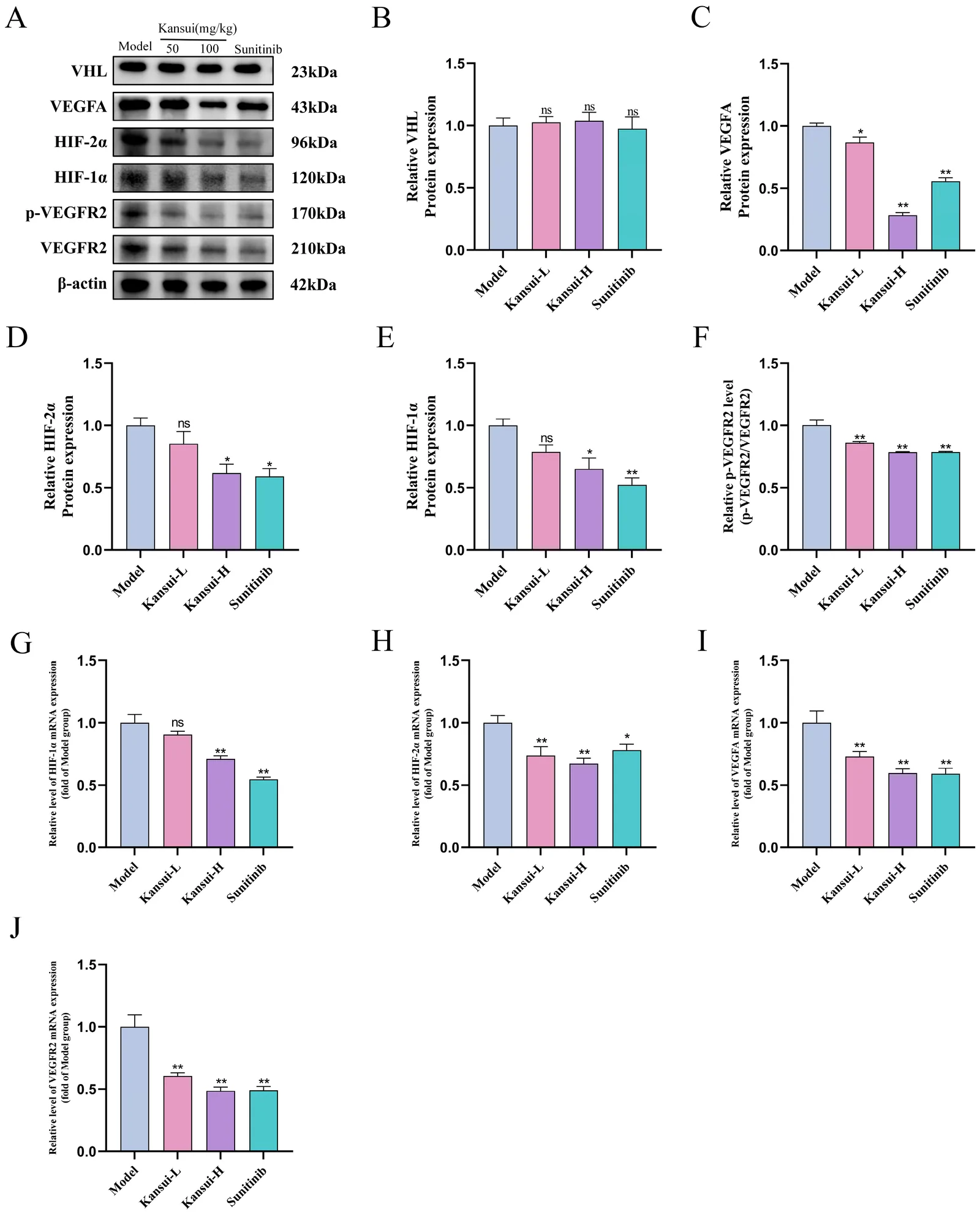
Effects of topically administered Kansui-DCM on the expression of VHL, HIF-1α, HIF-2α, VEGFA, and p-VEGFR2 proteins and expression of HIF-1α, HIF-2α, VEGFA, and VEGFR2 genes. (**A**–**F**) Expression of VHL, HIF-1α, HIF-2α, VEGFA, and p-VEGFR2 proteins, respectively; (**G**–**J**) expression of HIF-1α, HIF-2α, VEGFA, and VEGFR2 genes, respectively. All data are expressed as mean ± SD of three independent measurements (*n* = 3). Statistical significance was determined by one-way ANOVA with Dunnett’s post hoc test. * *p* < 0.05 and ** *p* < 0.01 vs. model group; “ns” means no significance.

*Von hippel–lindau* (*VHL*) gene alterations are associated with ccRCC, the predominant histological subtype of RCC [4]. As a tumor suppressor, VHL maintains HIF stability in normoxia, which in turn suppresses angiogenesis and subsequent tumor development [4]. Western blot analysis (Figure 8A–F) revealed no significant difference in VHL protein expression was observed between the model and Kansui-DCM groups (*p* > 0.05), suggesting that Kansui-DCM could not directly modulate VHL expression. In contrast, the expression levels of HIF-1α, HIF-2α, VEGFA, and phosphorylated VEGFR2 were significantly downregulated in both the low- and high-dose Kansui-DCM groups, exhibiting a clear dose-dependent effect. It is therefore plausible that the antitumor activity of Kansui-DCM might be mediated through the regulation of other key components within the HIF/VEGF signaling pathway.

### 2.9. Effects of Kansui-DCM on HIF/VEGF Signaling Receptors in a RENCA Subcutaneous Xenograft Mouse Model

RT-qPCR was conducted to gain deeper insight into the mechanisms driving the antitumor activity of Kansui-DCM in RCC-bearing mice. A significant reduction in the mRNA expression of *HIF-2α*, *VEGFA*, and *VEGFR2* was observed in the low-dose Kansui-DCM group, as compared with the model group (Figure 8G–J) (*p* < 0.01). In the high-dose Kansui-DCM group, the expression levels of *HIF-1α*, *HIF-2α*, *VEGFA*, and *VEGFR2* were all significantly decreased relative to the model group (*p* < 0.01) by targeting non-VHL pathway elements.

### 2.10. Molecular Docking of Kansui-DCM Components with Key Proteins in the HIF/VEGR Signal Pathway

The interactions between the 20 active components of Kansui-DCM and the key targets HIF-2α and VEGFR2 were assessed by molecular docking (Table 2). Binding energies below 0 kcal/mol signify that the ligand can bind the receptor spontaneously. A threshold of −5.0 kcal/mol is often taken to indicate favorable binding, whereas binding is considered strong when energies fall below −7.0 kcal/mol [25]. As presented in Table 3, the docking affinities of the tested compounds for VEGFR2 and HIF-2α exhibited marked differences, with an overall stronger binding propensity toward VEGFR2 than toward HIF-2α. Among them, 2,3-dihydrosciadopitysin exhibited the strongest binding affinity for VEGFR2 (−9.037 kcal/mol) while also maintaining a relatively strong affinity for HIF-2α. Notably, this compound displayed a balanced dual-target binding profile. Consequently, 2,3-dihydrosciadopitysin and 7-methoxy-3-[1-(3-pyridyl)methylidene]-4-chromanone were selected for subsequent interaction analysis and molecular dynamics simulations.

**Figure 9:**
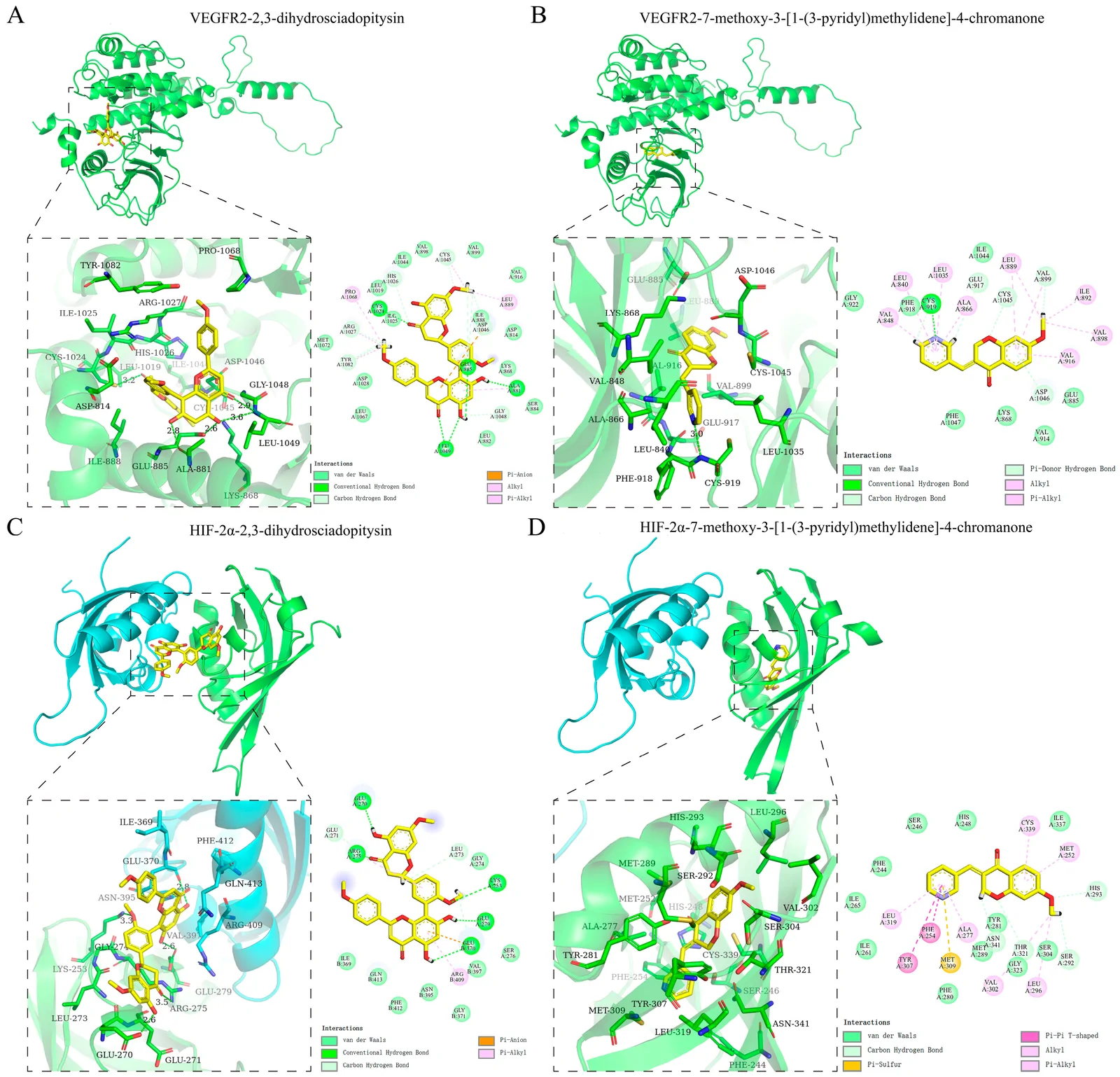
Docking results of VEGFR2/HIF-2α with 2,3-dihydrosciadopitysin and 7-methoxy-3-[1-(3-pyridyl)methylidene]-4-chromanone. (**A**) Docking results of VEGFR2 with 2,3-dihydrosciadopitysin; (**B**) docking results of VEGFR2 with 7-methoxy-3-[1-(3-pyridyl)methylidene]-4-chromanone; (**C**) docking results of HIF2α with 2,3-dihydrosciadopitysin; (**D**) docking results of HIF2α with 7-methoxy-3-[1-(3-pyridyl)methylidene]-4-chromanone.

The interaction modes of 2,3-dihydrosciadopitysin and 7-methoxy-3-[1-(3-pyridyl)methylidene]-4-chromanone with VEGFR2 and HIF-2α were analyzed using Discovery Studio and PyMOL v2.6. As shown in Figure 9A, 2,3-dihydrosciadopitysin formed hydrogen bonds with CYS1024, LEU1049, GLU665, and ALA881 of VEGFR2; hydrophobic interactions with PRO1068, ARG1027, TYR1082, ALA881, LEU889, and CYS1045; carbon–hydrogen bonds with HIS1026, ARG1027, TYR1082, GLY1048, GLU885, ASP1046, and CYS1045; a pi–anion interaction with ASP1046; and van der Waals contacts with surrounding residues. 7-Methoxy-3-[1-(3-pyridyl)methylidene]-4-chromanone formed a hydrogen bond with CYS919 of VEGFR2; hydrophobic interactions with VAL848, LEU840, LEU1035, ALA866, LEU889, ILE892, VAL898, and VAL916; carbon–hydrogen bonds with CYS919, ALA866, CYS1045, and VAL899; a pi–donor hydrogen bond with ASP1046; and van der Waals contacts with adjacent residues (Figure 9B).

With respect to HIF-2α, 2,3-dihydrosciadopitysin established hydrogen bonds with ARG275, GLU70, LYS253, GLU279, and GLU370; hydrophobic interactions with ARG275 and ARG409; carbon–hydrogen bonds with GLU271, LEU273, and GLU370; a pi–anion interaction with GLU370; and van der Waals contacts with surrounding amino acids (Figure 9C). In contrast, 7-methoxy-3-[1-(3-pyridyl)methylidene]-4-chromanone formed no hydrogen bonds with HIF-2α, but engaged in hydrophobic interactions with CYS339, MET252, LEU296, VAL302, ALA277, and LEU319; carbon–hydrogen bonds with HIS293, SER292, THR321, and ASN341; pi–pi T-shaped interactions with TYR307 and PHE254; a pi–sulfur interaction with MET309; and van der Waals contacts with neighboring residues (Figure 9D).

The smaller candidate compounds were predominantly located within the canonical binding cavities of HIF-2α and VEGFR2 and showed partial spatial overlap with the corresponding co-crystallized ligand-binding regions. In contrast, several structurally larger compounds occupied a broader region of the predefined binding pocket and extended into adjacent subpockets. These differences were likely related to variations in molecular size, geometry, and conformational flexibility.

Although the selected poses showed favorable docking scores and plausible interactions with residues surrounding the predicted binding regions, the absence of formal redocking validation limits the confidence of the predicted binding modes, particularly for the larger compounds. Therefore, these findings indicate potential structural compatibility rather than experimentally confirmed binding or inhibition.

### 2.11. Molecular Dynamics Simulations of the Interactions Between Kansui-DCM Components and Key HIF/VEGF Pathway Proteins

**Figure 10:**
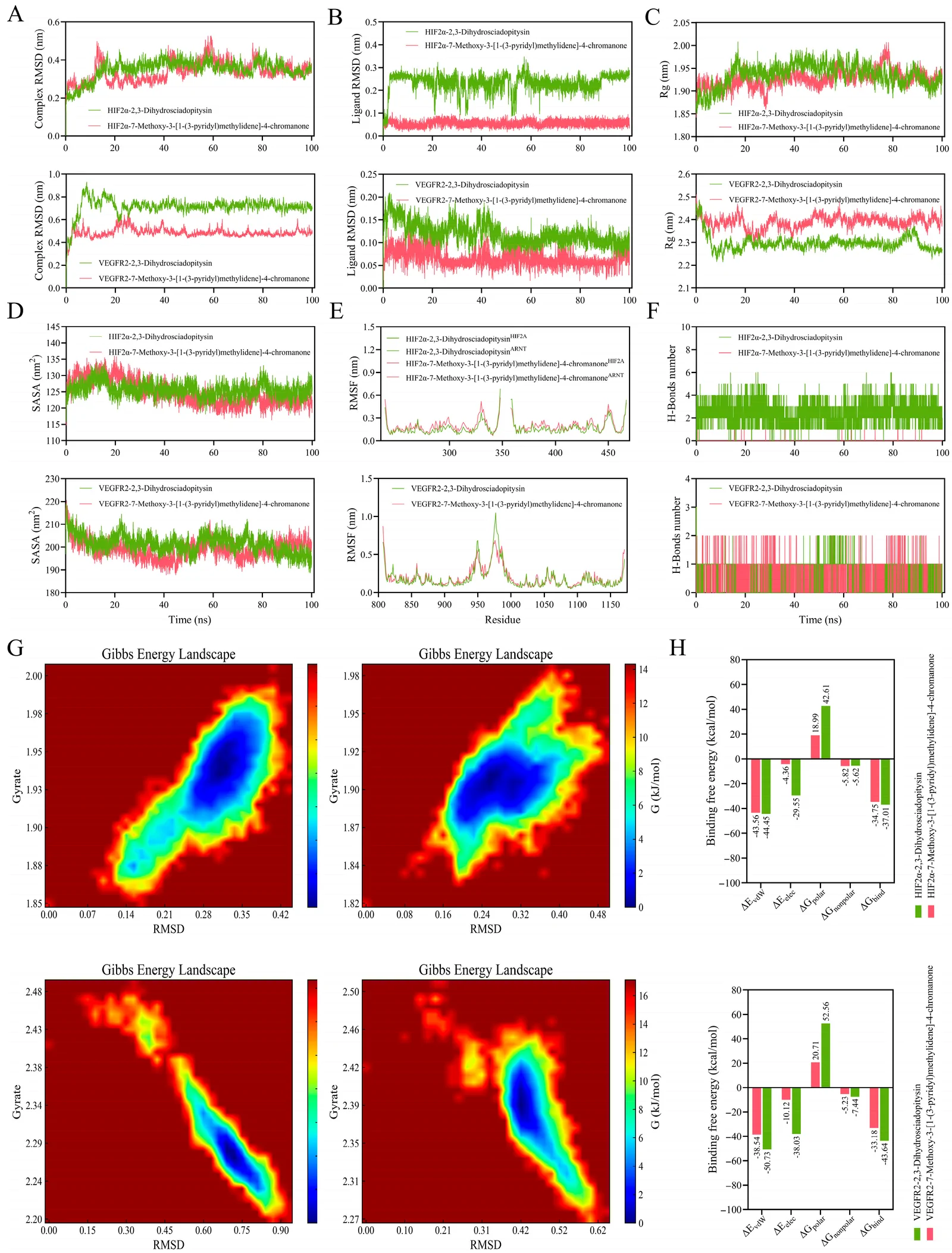
The 100 ns molecular dynamics simulation of the HIF-2α complex and VEGFR2 complex. (**A**) RMSD of the complexes; (**B**) RMSD of the ligands; (**C**) Rg of the proteins; (**D**) SASA of the proteins; (**E**) RMSF of the proteins; (**F**) hydrogen bond numbers of the complexes; (**G**) Gibbs free-energy landscape; (**H**) binding free-energy decomposition.

The molecular docking results guided subsequent 100 ns molecular dynamics simulations, which evaluated the dynamic stability of the small-molecule–protein complexes. Root-mean-square deviation (RMSD) was employed to evaluate the structural precision and overall stability of the simulated systems. As shown in Figure 10A, all four complexes underwent initial conformational relaxation before reaching a stable equilibrium. The HIF-2α–2,3-dihydrosciadopitysin complex stabilized at an RMSD of 0.33–0.40 nm, whereas the HIF-2α–7-methoxy-3-[1-(3-pyridyl)methylidene]-4-chromanone complex was maintained at 0.30–0.38 nm, with neither system exhibiting sustained drift or large-scale dissociation. In the VEGFR2 complexes, the RMSD of the 2,3-dihydrosciadopitysin-bound system fluctuated within a broader range of 0.65–0.78 nm, while that of the 7-methoxy-3-[1-(3-pyridyl)methylidene]-4-chromanone-bound system remained stable at 0.45–0.55 nm. Ligand RMSD analysis (Figure 10B) further revealed that 2,3-dihydrosciadopitysin displayed RMSD values of 0.20–0.30 nm and 0.09–0.13 nm within the binding pockets of HIF-2α and VEGFR2, respectively, with occasional transient fluctuations. In contrast, 7-methoxy-3-[1-(3-pyridyl)methylidene]-4-chromanone maintained a markedly narrow ligand RMSD range of 0.04–0.08 nm in both proteins, indicating a highly constrained binding orientation throughout the simulation.

The structural compactness of the complexes was evaluated via the radius of gyration (Rg), with a decrease in Rg signifying tighter folding (Figure 10C). In the HIF-2α complexes, the Rg values of both systems primarily ranged from 1.90 to 1.96 nm. The 2,3-dihydrosciadopitysin-bound complex exhibited an initial increase from approximately 1.87 nm to 1.94 nm before reaching a plateau, whereas the 7-methoxy-3-[1-(3-pyridyl)methylidene]-4-chromanone-bound complex displayed a more concentrated Rg distribution with a narrower fluctuation range. In the VEGFR2 complexes, the Rg values of the 2,3-dihydrosciadopitysin system primarily ranged from 2.26 to 2.32 nm, which were lower than those of the 7-methoxy-3-[1-(3-pyridyl)methylidene]-4-chromanone system (2.35–2.43 nm), suggesting that the former adopted a more compact conformation while the latter was comparatively less compact.

The solvent-accessible surface area (SASA) was evaluated to assess the exposure of protein residues to the surrounding solvent (Figure 10D). In the HIF-2α complexes, the SASA of the 2,3-dihydrosciadopitysin-bound system remained in the range of 123–129 nm^2^, whereas that of the 7-methoxy-3-[1-(3-pyridyl)methylidene]-4-chromanone-bound system gradually decreased from 128–133 nm^2^ to 119–123 nm^2^, indicating the adoption of a more compact conformation. In the VEGFR2 complexes, the SASA of 2,3-dihydrosciadopitysin primarily ranged from 190 to 205 nm^2^, with a slight decrease in the later stage of the simulation, while that of 7-methoxy-3-[1-(3-pyridyl)methylidene]-4-chromanone ranged from 193 to 205 nm^2^ and was marginally higher at the end of the simulation, suggesting that the former maintained a lower solvent-exposed surface area.

Root-mean-square fluctuation (RMSF) analysis was employed to monitor the conformational flexibility of individual residues throughout the simulation, where smaller values denoted higher local stability. In both complexes, the majority of residues showed low RMSFs (mainly 0.10–0.30 nm), indicative of overall backbone stability (Figure 10E). In the HIF-2α complexes, elevated fluctuations were primarily observed near residues 340–355 and around residue 450; notably, the 2,3-dihydrosciadopitysin-bound system displayed a more prominent peak at approximately residue 350, reaching a maximum RMSF of 1.3–1.4 nm, whereas the 7-methoxy-3-[1-(3-pyridyl)methylidene]-4-chromanone-bound system exhibited lower fluctuations in this region. In the VEGFR2 complexes, pronounced fluctuation peaks were concentrated around residues 950–990, with peak values of 0.7–1.0 nm; the overall fluctuation profiles of the two systems were highly similar, although the 7-methoxy derivative showed slightly elevated fluctuations at certain local peaks.

Intermolecular hydrogen bonds were evaluated as an indicator of complex stability throughout the simulation. Analysis of the intermolecular hydrogen bonds revealed distinct interaction patterns between the two ligands and their respective receptors (Figure 10F). In the HIF-2α complexes, 2,3-dihydrosciadopitysin consistently formed a greater number of hydrogen bonds, predominantly ranging from 2 to 5, with occasional peaks reaching 6. In contrast, the 7-methoxy-3-[1-(3-pyridyl)methylidene]-4-chromanone-bound system maintained only 0–1 hydrogen bonds during most of the simulation. In the VEGFR2 complexes, both ligands formed relatively few hydrogen bonds, with 0–1 hydrogen bonds maintained for the majority of the simulation and only occasional peaks of 2, indicating the absence of a persistent and extensive hydrogen bond network.

Free-energy landscape (FEL) analysis revealed that all four complexes formed well-defined low-energy conformational states (Figure 10G). In the HIF-2α system, 2,3-dihydrosciadopitysin exhibited a deep and narrow energy basin centered at an RMSD of 0.28–0.35 nm and an Rg of 1.92–1.97 nm. In contrast, 7-methoxy-3-[1-(3-pyridyl)methylidene]-4-chromanone displayed a primary low-energy region at an RMSD of 0.22–0.33 nm and an Rg of 1.88–1.92 nm, accompanied by metastable distributions extending toward higher RMSD and Rg values. In the VEGFR2 system, 2,3-dihydrosciadopitysin favored a more compact conformation with the global minimum located in the lower Rg region (RMSD 0.65–0.82 nm, Rg 2.22–2.31 nm), whereas the 7-methoxy derivative exhibited a primary energy minimum shifted toward a lower RMSD range (RMSD 0.40–0.50 nm, Rg 2.35–2.42 nm).

MM/PBSA calculations demonstrated that both ligands formed stable complexes with HIF-2α and VEGFR2, albeit with distinct thermodynamic profiles (Figure 10H). In the HIF-2α system, 2,3-dihydrosciadopitysin exhibited a more favorable total binding free energy (−37.01 kcal/mol) compared with 7-methoxy-3-[1-(3-pyridyl)methylidene]-4-chromanone (−34.75 kcal/mol). Both complexes were predominantly stabilized by van der Waals interactions; however, the former displayed a more pronounced electrostatic contribution (−29.55 kcal/mol) despite a high polar solvation energy penalty (42.61 kcal/mol). Similarly, in the VEGFR2 system, 2,3-dihydrosciadopitysin showed more favorable binding free energy (−43.64 kcal/mol vs. −33.18 kcal/mol), with stronger van der Waals (−50.73 kcal/mol) and electrostatic (−38.03 kcal/mol) contributions, which outweighed the higher polar solvation penalty (52.56 kcal/mol).

## 3. Discussion

Advances in ICI-based therapies and surgery have improved RCC outcomes [26], but challenges remain in biomarker validation, the role of surgery, and gaps in the understanding of non-clear cell and hereditary RCC [27]. Kansui shows anticancer potential, but its mechanisms remain unclear and comprehensive studies on its anticancer effects are still limited [13,28]. The anti-RCC efficacy of Kansui and its mechanisms were explored in this study. The experiments utilized 786-O cells, a human clear cell RCC line harboring VHL mutation, and RENCA cells, a murine spontaneous RCC line appropriate for allograft models—both commonly adopted in renal cancer research for in vitro and in vivo work [29,30]. RENCA cells, derived from a spontaneous renal adenocarcinoma in BALB/c mice, are widely used to generate orthotopic or subcutaneous RCC models. These models facilitate the study of tumor biology, immune microenvironment dynamics, and the effectiveness of combination immunotherapies, such as immune checkpoint inhibitors and TLR agonists [31,32]. In this study, Kansui Radix was tested for its anti-RCC activity in vitro on 786-O and RENCA cells and in vivo using a subcutaneous RENCA mouse model.

Kansui Radix has been reported to produce steroids targeting ATP1A1 for liver cancer [9], kidjolanin targeting STAT3/EGFR for lung cancer [13], and diterpenoids that reverse multidrug resistance [12]. This work focused on the mechanisms underlying the anti-RCC activity of Kansui Radix. Our findings suggest that the antitumor activity of Kansui Radix in renal cell carcinoma warrants further investigation. Proliferation of cancer cells is regarded as a central factor in tumor growth and disease progression [33]. CCK-8 assays were performed at various time points to examine how Kansui-DCM extracts inhibited the proliferation of 786-O and RENCA cells. The results indicated that Kansui-DCM exhibited significant, dose-dependent inhibitory effects on both 786-O and RENCA cells.

The process of apoptosis, or programmed cell death, involves a series of biochemical reactions culminating in distinct morphological changes such as cell shrinkage, condensed chromatin, and fragmented nuclear DNA [34]. The induction of apoptotic cell death is critical to the therapeutic action of numerous antitumor agents [35]. Hoechst staining detects condensed DNA and thereby separates apoptotic cells from healthy or necrotic populations [36]. The Annexin V-FITC/PI assay distinguishes apoptotic from viable and necrotic cells by exploiting Annexin V’s binding to externalized phosphatidylserine and PI’s staining of membrane-compromised cells, thereby enabling flow cytometric classification into viable, early-apoptotic, late-apoptotic, and dead populations [37]. Hoechst staining and flow cytometry showed that Kansui-DCM dose-dependently increased apoptosis in both 786-O and RENCA cells, supporting its therapeutic potential against RCC. The consistent pro-apoptotic effect observed by two independent methods strengthens the conclusion that Kansui-DCM triggers apoptotic cell death rather than simple cytotoxicity.

The malignant behavior of progressing tumors is largely attributable to metastasis, which fundamentally depends on the processes of migration and invasion [38]. The Transwell assay quantitatively evaluates tumor cell chemotaxis by assessing migration through a membrane toward a chemoattractant gradient; coating the membrane with extracellular matrix components allows this assay to also measure invasive potential [39,40]. Our results showed that Kansui-DCM significantly impaired the migratory and invasive abilities of RENCA cells. However, this effect was cell-line-dependent, as 786-O cells showed no genuine inhibition after normalization, suggesting that the anti-metastatic potential of this extract may vary across RCC subtypes. Therefore, Kansui may possess the potential to suppress renal carcinoma metastasis, a finding that aligns with its traditional use for “dissipating masses” (zheng-jia).

CD8^+^T cells are cytotoxic effectors whose density in tumor regions predicts patient survival [41]. CD31 is an endothelial cell marker used to quantify microvessel density (MVD) as a surrogate for tumor angiogenic activity [42]. Ki67 is a nuclear protein employed to assess tumor cell proliferation and serves as a prognostic marker in early breast cancer [43]. COX-2 converts arachidonic acid into prostaglandins, thereby promoting inflammation and cell proliferation [44]. Our results demonstrated that Kansui-DCM reduced the expression of CD31, Ki67, and COX-2 while enhancing CD8+ T-cell expression, suggesting that Kansui exerts its therapeutic potential by inhibiting tumor tissue growth and remodeling the immune microenvironment toward an antitumor state. The increase in CD8^+^ T-cell infiltration is particularly noteworthy, as it implies that Kansui-DCM might potentiate host antitumor immunity, an effect that could be exploited in combination with immune checkpoint inhibitors.

Transcriptomic analysis of mouse tumor tissues was carried out via high-throughput sequencing to investigate how Kansui-DCM exerts its antitumor effects in RCC-bearing mice. The screening results revealed 130 overlapping targets between the genes identified by WGCNA and the drug components detected via UPLC-MS. The pathways involving these overlapping targets included the EGFR signaling pathway, PI3K-Akt signaling pathway, TNF signaling pathway, and VEGF signaling pathway, among others. VEGF drives tumor angiogenesis by specifically promoting endothelial cell growth, making it a key anticancer therapeutic target [45]. HIFs regulate gene transcription in response to hypoxia [20], driving VEGF expression to promote angiogenesis [46,47]. These transcriptomic findings, together with the KEGG enrichment results, pointed to the HIF/VEGF pathway as a candidate mechanism potentially mediating Kansui-mediated RCC suppression. However, it is important to emphasize that these bioinformatic predictions are correlative in nature and should be interpreted as hypothesis-generating rather than as demonstrated pathway engagement. Subsequently, we performed in vivo experiments to validate whether the inhibitory effect of Kansui-DCM on renal cell carcinoma is associated with the HIF/VEGF pathway. Protein immunoblotting results indicated that in tumor tissues of RCC mice without VHL gene knockout, the tumor microenvironment induces hypoxia, which promotes cancer cell adaptation and tumor progression [48]. HIF-1α accumulated in the model group due to the influence of the tumor microenvironment. Compared with the model group, the expression levels of HIF-1α and HIF-2α in the treatment groups were significantly reduced. Furthermore, under hypoxic conditions within the tumor microenvironment, the expression levels of both VEGFA and VEGFR2 proteins were higher in the model group than in the drug-treated group. These results suggested that Kansui-DCM treatment is associated with the downregulation of HIF/VEGF signaling, which may contribute to its antitumor effects. qPCR results also indicated that Kansui treatment significantly suppresses the HIF/VEGF pathway. Of particular interest is that VHL protein levels remained unchanged after Kansui-DCM treatment in the RENCA model (which is VHL-wild-type), suggesting that the suppression of HIF/VEGF signaling may occur independently of VHL protein expression. However, as RENCA cells are not VHL-deficient, this observation does not establish a VHL-independent mechanism in the context of ccRCC; future studies in VHL-deficient models would be required to test this possibility. This is clinically relevant because the majority of clear cell RCCs harbor inactivating VHL mutations; agents that suppress HIF-α independently of VHL may overcome a key resistance mechanism associated with VHL loss [49]. Several upstream regulators could be involved, including the PI3K/Akt/mTOR axis, reactive oxygen species (ROS) accumulation [50], or modulation of HIF-α hydroxylase activity by non-VHL pathways [51]. Future studies should explore these possibilities.

Molecular docking coupled with molecular dynamics simulations jointly provided computational predictions of the differential binding mechanisms of 2,3-dihydrosciadopitysin and 7-methoxy-3-[1-(3-pyridyl)methylidene]-4-chromanone to VEGFR2 and HIF-2α from the perspectives of both static interaction patterns and dynamic stability characteristics. Docking analysis predicted that 2,3-dihydrosciadopitysin formed a more extensive network of polar interactions in both targets, including multiple hydrogen bonds and pi–anion interactions with residues such as Cys1024 and Glu665 of VEGFR2 and Arg275 and Glu370 of HIF-2α, with a broader distribution of C–H hydrogen bonds. In contrast, the 7-methoxy derivative exhibited relatively limited polar interactions and, notably, formed no hydrogen bonds with HIF-2α; its binding was primarily driven by hydrophobic interactions and non-classical contacts such as π-π π−π T-shaped stacking and pi–sulfur interactions. These static features were highly consistent with the dynamic behavior: 2,3-dihydrosciadopitysin achieved a more favorable MM/PBSA binding free energy owing to stronger electrostatic and van der Waals contributions; however, its abundant dynamic hydrogen bond network was accompanied by higher ligand RMSD fluctuations and local RMSF peaks, suggesting that its multiple polar contacts may confer a certain degree of conformational flexibility within the binding pocket. In contrast, the 7-methoxy derivative, despite fewer polar contacts, exhibited extremely low ligand RMSD values (0.04–0.08 nm) in both targets and displayed a more converged low-RMSD energy basin in the free-energy landscape, indicating that its stable anchoring relies more on non-hydrogen-bonding interactions such as shape complementarity, hydrophobic matching, and π-related stacking. This interaction mode endows the complex with stronger spatial constraint and conformational retention. Overall, our computational predictions suggested that 2,3-dihydrosciadopitysin may possess a superior thermodynamic driving force and a rich polar interaction network, whereas 7-methoxy-3-[1-(3-pyridyl)methylidene]-4-chromanone may offer advantages in structural stability and spatial anchoring precision. Taken together, these two compounds highlight their potential as candidate inhibitors of VEGFR2 and HIF-2α from complementary dimensions.

Our findings demonstrate that Kansui-DCM inhibits tumor angiogenesis, providing a molecular basis for reducing renal mass formation. Moreover, the immunomodulatory effect on CD8^+^ T lymphocytes suggests that immune activation contributes critically to the regression of pathological masses. Thus, this work establishes a pharmacological foundation for the anti-renal cell carcinoma activity of Kansui Radix, linking its anti-angiogenic and immunomodulatory effects to tumor inhibition.

Current research findings provided a robust research foundation and data support for the anti-renal cancer effects of Kansui Radix. Nevertheless, several limitations should be acknowledged. First, the RENCA allograft model is VHL-wild-type and does not recapitulate the VHL-deficient genetic background that drives the majority of human ccRCC cases. Our findings should be interpreted as pharmacological modulation of HIF/VEGF pathway components rather than direct evidence of efficacy against VHL-driven ccRCC. Second, the individual contributions of the two prioritized compounds to the in vivo antitumor effect remain to be quantified, and Kansui-DCM is a complex non-standardized fraction, making attribution of observed effects to specific constituents challenging. Third, in vivo toxicity evaluation was limited to body weight monitoring and general observation; serum biochemical parameters (e.g., ALT, AST, BUN, Cr) and histopathological examination of major organs were not performed. Additionally, the known toxicity of Kansui Radix to the kidney, liver and gastrointestinal tract requires long-term safety evaluation before clinical translation. Fourth, unchanged VHL protein levels do not exclude possible effects on VHL functional activity, which was not directly assessed. Fifth, changes in HIF/VEGF pathway components are correlative rather than causal, as pathway-specific rescue or gene manipulation experiments were not performed. Sixth, in vitro cytotoxicity was evaluated only in tumor cell lines, without parallel assessment in normal renal epithelial cells. Seventh, molecular validation of apoptosis pathway activation (e.g., Bcl-2, Bax, cleaved caspase-3) was not performed. Eighth, the docking protocol was not validated by redocking the co-crystallized ligands, and docking scores and MD stability are computational estimates that do not establish biological inhibition in cells or tumors.

Despite these limitations, the present study provided compelling multi-level evidence that Kansui-DCM inhibits RCC progression via the HIF/VEGF pathway, paving the way for future mechanistic dissection of VHL-independent HIF regulation and clinical translation of this herbal fraction. Additionally, the observed cell-line-dependent anti-metastatic effects warranted further validation in additional RCC models. Therefore, we concluded that Kansui Radix holds significant promise as a drug candidate for renal cell carcinoma research, and addressing the above-mentioned limitations in future investigations will be essential to advance this traditional medicine toward clinical application.

## 4. Materials and Methods

### 4.1. Chemicals and Reagents

The roots of *Euphorbia kansui* T. N. Liou ex S. B. Ho (called Kansui Radix) were purchased from Hanzhong, Shaanxi province. The identification of this plant was carried out by Prof. Jianping Gao (Shanxi Medical University, Shanxi, China). The voucher specimen (No. 20240701) has been archived at the Department of Traditional Chinese Medicine, Shanxi Medical University. Sunitinib was purchased from GlpBio (Cat. GC17651). Gibco (Grand Island, NY, USA) supplied the RPMI-1640 medium and penicillin–streptomycin, while fetal bovine serum (FBS) was sourced from Wuhan Pricella Biotechnology Co., Ltd. (Wuhan, China).

### 4.2. Preparation and Extraction of Kansui Radix

Kansui Radix was pulverized and extracted three times with 80% ethanol under reflux heating for 1.5 h each time. After combination, the extracts were concentrated by rotary evaporation, and the resulting concentrate was collected. The extract was dispersed in water and then partitioned with dichloromethane, ethyl acetate, and finally n-butanol. After vacuum concentration, the dichloromethane fraction, ethyl acetate fraction, and n-butanol fraction were obtained [52].

### 4.3. Cell Lines and Culture

786-O (human renal clear cell adenocarcinoma cells) and RENCA (mouse renal carcinoma) cells were acquired from Abbkine and cultured in RPMI-1640 supplemented with 10% heat-inactivated FBS and 1% streptomycin/penicillin in a humidified atmosphere with 5% CO_2_ at 37 °C.

### 4.4. CCK-8 Assay

Cell viability was determined by Cell Counting Kit-8 (CCK-8) assay. 786-O cells and RENCA cells were seeded into 96-well plates at a density of 1 × 10^4^ cells/well and cultured until reaching 80–90% confluence. Subsequently, the dichloromethane, ethyl acetate, or n-butanol extracts of Kansui (designated as Kansui-DCM, ethyl acetate fraction, and n-butanol fraction) at concentrations of 0, 5, 10, 20, 40, 60, 80, 100 μg/mL were added to these cells. Following a 24 h culture period, CCK-8 reagent was applied, and the cells were held at 37 °C for another 1 h. A microplate reader was used to record the optical density (OD) at 450 nm. The experiment was repeated three times for statistical analysis.

### 4.5. Chromatographic Analysis and UPLC-MS Identification of Kansui-DCM

Samples were thawed from −80 °C, extracted with pre-chilled 70% methanol containing the internal standard at a volume of 600 µL per 50 mg sample, vortexed for 15 min, centrifuged (12,000 rpm, 4 °C, 3 min), and filtered (0.22 µm) prior to LC-MS/MS. The internal standard was used to correct for extraction and injection variability. A pooled quality-control (QC) sample was prepared by mixing equal aliquots of all samples and analyzed alongside the test samples to monitor system stability. Since authentic standards were unavailable for the majority of the detected compounds, all identifications are considered putative, with confidence levels classified into three grades (Level 1 to Level 3) based on the consistency of MS/MS spectral matching and retention time with the MWDB database. The specific identification level for each compound is provided in Table 1. According to the MWDB matching criteria: Level 1, MS/MS spectrum (all fragment ions) and retention-time match score ≥ 0.7; Level 2, MS/MS spectrum and retention-time match score 0.5–0.7; and Level 3, Q1, Q3, retention time, DP, and CE matched the database entry without full MS/MS confirmation. The LC-MS/MS platform included an ExionLC™ AD UPLC (SCIEX) and a triple quadrupole mass spectrometer. Chromatography was conducted on an Agilent SB-C18 column (1.8 μm, 2.1 × 100 mm) kept at 40 °C, with 0.1% formic acid in water (A) and 0.1% formic acid in acetonitrile (B) as the mobile phases. The gradient profile was as follows: 0–9 min, 5% to 95% B; 9–10 min, 95% B; at 10 min, return to 5% B, hold for 1.1 min, and re-equilibration at 5% B until 14 min. The flow rate and injection volume were set to 0.35 mL/min and 2 μL, respectively. ESI source parameters were as follows: temperature, 500 °C; ion-spray voltage, +5500 V/−4500 V; GS1, 50 psi; GS2, 60 psi; CUR, 25 psi; CAD gas, high. Multiple reaction monitoring (MRM) was performed with medium collision gas (nitrogen). The declustering potential (DP) and collision energy (CE) were tailored to each MRM transition. In each time segment, a specific set of transitions was selected for monitoring based on the metabolites eluting at that time. Before each batch, a standard mixture was injected to verify column performance, ensuring retention-time drift <0.2 min. Metabolite identification was further performed by matching MS/MS spectra against the MWDB database, using retention time, precursor mass, and fragment ions. All quantitation is relative: peak areas represent relative abundance for cross-sample comparisons; absolute concentrations are not reported (no calibration curves were constructed). According to the data preprocessing criteria, metabolites with a coefficient of variation (CV) ≥ 0.5 across QC injections were excluded, retaining only those with CV < 0.5 for subsequent analysis. The extraction protocol (70% methanol at −20 °C) is a widely established method in the metabolomics field; although the absolute extraction recovery was not independently determined, the inclusion of an internal standard together with QC-based CV filtering effectively compensates for potential losses during extraction, ensuring the reliability of relative comparisons.

### 4.6. Morphological Observation

Hoechst 33,258 staining assay (Beyotime Biotechnology, Shanghai, China) was performed to observe the morphological characteristics of apoptotic cells [53]. 786-O and RENCA cells were seeded into a 6-well plate at a density of 3 × 10^5^ cells/well. 786-O cells were exposed to Kansui-DCM at 0, 25, 50, or 75 μg/mL and RENCA cells to 0, 10, 25, or 50 μg/mL for 24 h. After treatment, we fixed the cells with 4% paraformaldehyde for 20 min, rinsed them three times with PBS, and then stained them with 500 μL Hoechst 33,258 (10 μg/mL) for 5 min in the dark, followed by a final PBS wash. Nuclear morphology was examined under a Leica DMi8 fluorescence microscope. Three independent experiments were carried out.

### 4.7. Annexin V-FITC/PI Analysis

An Annexin V-FITC/PI Apoptosis Detection Kit (Dalian Meilune, Dalian, China) was employed for apoptosis measurement, and the assay was conducted according to the supplier’s guidelines. 786-O and RENCA cells were plated at 3 × 10^5^ cells/well in 6-well plates and subsequently exposed to Kansui-DCM for 24 h at concentrations of 0, 25, 50, 75 μg/mL (786-O) and 0, 10, 25, 50 μg/mL (RENCA). Treated cells were trypsinized (EDTA-free), washed in cold PBS, and taken up in 200 μL binding buffer. Annexin V-FITC (5 μL) and PI (5 μL) were added, and the mixture was left for 30 min in the dark. It was then strained (100 μm mesh), mixed with 400 μL binding buffer, and run on the flow cytometer within 1 h. Experiments were repeated three times.

### 4.8. Transwell Assay

RENCA cell migration (uncoated) and invasion (Matrigel-coated) were examined using 8.0 μm pore Transwell filters (Corning). Based on the cytotoxicity profiles determined by parallel 24 h CCK-8 assays, cells were treated with Kansui-DCM at non-cytotoxic (10 μg/mL) and minimally cytotoxic (20 μg/mL) concentrations. Kansui-DCM-treated cells (4 × 10^4^) were resuspended in 200 μL serum-free medium containing the corresponding drug concentration and introduced into the upper chamber, while 500 μL of complete medium (20% FBS) with the same drug concentration was added to the lower compartment. Incubation proceeded for 24 h at 37 °C. Following incubation, the undersurface cells were subjected to fixation in 4% paraformaldehyde and staining with 0.1% crystal violet. Stained cells were counted in at least three randomly selected fields per chamber under a microscope. All data were normalized to the corresponding cell viability determined by parallel CCK-8 assays. Each concentration was tested in triplicate chambers, and the experiment was repeated three times independently.

### 4.9. Animals

Five-week-old male Balb/c mice (20 ± 2 g), purchased from Beijing Huafukang Biotechnology Co., Ltd. (Beijing, China), were housed at the Experimental Animal Center of the School of Pharmacy, Shanxi Medical University, under specific pathogen-free conditions at 22–25 °C and 55–65% relative humidity, with a 12 h light/dark cycle and free access to food and water. All animal experiments were approved by the Institutional Animal Care and Use Committee of Shanxi Medical University and performed in accordance with the ARRIVE guidelines.

### 4.10. Modeling and Drug Delivery

After one week of adaptation, mice were subcutaneously inoculated in the right axilla with 1 × 10^6^ RENCA cells suspended in 100 μL PBS to establish tumor-bearing models. Tumor dimensions were measured every two days using a digital caliper, and tumor volume was calculated according to the formula: V = (length × width^2^) × 0.5. At a tumor volume of 50–100 mm^3^, mice were stratified by tumor size and randomly assigned to four groups (*n* = 6 per group) using a random number table: vehicle-treated model, Kansui-L (50 mg/kg), Kansui-H (100 mg/kg), and sunitinib (20 mg/kg). Kansui-DCM was suspended in 0.5% carboxymethylcellulose sodium (CMC-Na) and administered once daily by oral gavage at a volume of 10 μL/g body weight for 14 consecutive days. Sunitinib was dissolved in the same vehicle and administered with the same regimen. The vehicle control group received an equal volume of 0.5% CMC-Na. Tumor measurements and body weight recordings were performed by an investigator blinded to the treatment assignments, while drug administration was performed by a separate investigator. Mice were euthanized by cervical dislocation under isoflurane anesthesia when tumor volume exceeded 1500 mm^3^ or when signs of severe distress (e.g., body weight loss > 20%, lethargy, or tumor ulceration) were observed. Animals that failed to develop tumors or died from causes unrelated to treatment were excluded from analysis. A tumor-free normal cohort (*n* = 6) receiving vehicle alone was maintained in parallel for histopathological comparison. After 14 days of treatment, all surviving mice were euthanized, and tumor tissues were excised from mice in all tumor-bearing groups (model, Kansui-L, Kansui-H, and sunitinib) and weighed.

### 4.11. HE Staining

The tumors were fixed, paraffin-embedded, and sectioned at 5 μm. The resulting H&E-stained sections were observed microscopically to distinguish the morphological structure differences between the different treatment groups.

### 4.12. Immunohistochemistry (IHC) and Immunofluorescence (IF) Staining

Following deparaffinization, rehydration, and heat-induced antigen retrieval in citrate buffer (pH 6.0), the sections were treated with 3% H_2_O_2_ to quench endogenous peroxidase and subsequently blocked with 5% BSA (30 min, RT) [54]. The sections were then incubated overnight at 4 °C with primary antibodies against CD8 (Cat. No. ab237709), CD31 (Cat. No. ab182981), COX-2 (Cat. No. ab179800) and Ki-67 (Cat. No. ab15580) (all from Abcam, Waltham, MA, USA). Following rinsing, the sections were exposed to biotinylated secondary antibody (1 h, RT), and then visualized with DAB and hematoxylin counterstaining. Micrographs were taken on a Leica DMi8 microscope (Leica, Wetzlar, Germany), and the immunoreactive area was measured using ImageJ (Media Cybernetics, Inc., Rockville, MD, USA).

Cryostat sections (5 μm) of tumor tissue were subjected to blocking in 5% goat serum (1 h), individual overnight incubation with anti-VEGFA and anti-HIF-1α antibodies at 4 °C, PBS rinses, fluorescent secondary antibody labeling (1 h), and DAPI staining (10 min). Images were captured on a fluorescence microscope, and the immunoreactive area was quantified by ImageJ.

### 4.13. RNA Extraction and Sequencing

Total RNA was extracted from tumor tissues (*n* = 3 biologically independent samples per group: model, Kansui-L, Kansui-H, and sunitinib) using TRIzol^®^ reagent (Magen) according to the manufacturer’s instructions. RNA concentration and purity were assessed using a Nanodrop ND-2000 spectrophotometer (Thermo Fisher Scientific, Waltham, MA, USA), and RNA integrity was evaluated using an Agilent Bioanalyzer 4150 with the RNA 6000 Nano Kit. Only samples with an RNA Integrity Number (RIN) ≥ 7.0 and A_260_/A_280_ ratios between 1.8 and 2.1 were used for subsequent library construction [55]. Stranded mRNA libraries were constructed using the ABclonal mRNA-seq Lib Prep Kit following the manufacturer’s protocol. The libraries were sequenced on an Illumina Novaseq X Plus platform in paired-end mode, generating approximately 6–8 Gb of clean data per sample.

### 4.14. RNA-Seq Data Processing and Differential Analysis

Raw reads underwent adapter trimming and quality filtering using fastp with the following criteria: reads containing adapters, reads with >5% ambiguous bases (N), and low-quality reads (where the number of bases with quality value ≤25 exceeded 60% of the read length) were removed. The resulting clean reads were mapped to the mouse reference genome (GRCm38/mm10) by HISAT2 (v2.2.0) (http://daehwankimlab.github.io/hisat2/ accessed on 5 March 2025), with the mapping rate exceeding 85% for all samples. Gene-level read counts were generated with FeatureCounts (v2.0.0) (http://subread.sourceforge.net/ accessed on 7 March 2025). FPKM values were then calculated to quantify expression. Differential expression analysis between the model group and each treatment group was performed with DESeq2 (v1.38.0) (http://bioconductor.org/packages/release/bioc/html/DESeq2.html accessed on 10 March 2025). Principal component analysis (PCA) was performed to assess overall sample clustering. Genes were considered significantly differentially expressed (DEGs) when the |log_2_ fold change| > 1 and the adjusted *p*-value < 0.05, with the false-discovery rate controlled using the Benjamini–Hochberg method.

### 4.15. Weighted Gene Co-Expression Network Analysis (WGCNA)

WGCNA was conducted on all expressed genes (FPKM > 1 in at least 50% of samples) using the WGCNA R package (v1.71). A total of 24 samples (*n* = 6 per group: model, Kansui-L, Kansui-H, and sunitinib) were included in the analysis. Input data included transcriptome-derived genes and five phenotypic indicators (body weight, tumor weight, tumor volume, spleen index, and kidney index). Following preprocessing, the optimal soft-thresholding power (β = 5) was selected based on the scale-free topology fit index (R^2^ > 0.85) and mean connectivity. A co-expression network was constructed using the signed adjacency matrix, and genes were clustered into modules using average-linkage hierarchical clustering with the dynamic tree-cut algorithm (minimum module size = 30). Modules with similar expression profiles were merged to obtain the final set of co-expression modules. To identify phenotype-associated modules, Pearson’s correlation was calculated between module eigengenes and each phenotypic trait, with statistical significance set at *p* < 0.05.

### 4.16. Targets Related to the Active Ingredients in Kansui-DCM

The compounds related to Kansui-DCM were searched in the Traditional Chinese Medicine Systematic Pharmacology Database and Analysis Platform (TCMSP, https://www.tcmsp-e.com/#/home accessed on 10 March 2025). The OB (absorbability) ≥ 30% and DL (drug likeness) ≥ 0.18 combined were used as the screening conditions to screen out the main active ingredients. The main active compounds detected in Kansui-DCM by UPLC-MS were selected, and their putative targets were retrieved from the Swiss Target Prediction platform (http://www.swisstargetprediction.ch/ accessed on 10 March 2025).

### 4.17. GO and KEGG Pathway Enrichment Analysis

The overlapping genes between transcriptome-based WGCNA genes and UPLC-MS-guided component targets were predicted to be potential therapeutic targets of Kansui-DCM against RCC. Gene Ontology (GO) functional annotation and Kyoto Encyclopedia of Genes and Genomes (KEGG) pathway analysis were performed to systematically elucidate the biological functions of the potential therapeutic targets on https://www.bioinformatics.com.cn accessed on 10 March 2025. The GO enrichment results consisted of cellular component (CC), biological process (BP), and molecular function (MF) terms. The screening criteria for both analyses were set at *p*-value < 0.05 and false-discovery rate (FDR) < 0.1.

### 4.18. Quantitative Real-Time Polymerase Chain Reaction (qRT-PCR) Experiments

Total RNA was extracted from tumor tissues using the M5 Universal RNA Mini Kit (Mei5bio, Beijing, China) and then converted to cDNA with a cDNA Synthesis Supermix (Transgen, Beijing, China). Quantitative real-time PCR was performed on a Roche LightCycler 96 using 2× M5 HiPer SYBR Premix EsTaq (Mei5bio, Beijing, China). A 20 μL reaction consisted of 10 μL SYBR Premix, 2 μL cDNA, 7.2 μL nuclease-free water, and 0.4 μL of each primer. The temperature profile comprised 94 °C for 30 s and 40 cycles of 94 °C for 5 s/60 °C for 30 s. β-Actin served as the reference gene, and the primers were obtained from Tsingke (Beijing, China) (sequences in Table 3). Relative expression was determined with the 2^−^ΔΔCt 2^−ΔΔCt^ method.

### 4.19. Western Blot

Protein extraction from tumor tissues employed RIPA buffer with protease and phosphatase inhibitors, followed by BCA quantification. After resolution on 10% SDS-PAGE and transfer to PVDF membranes, the blots were blocked with 10% BSA and then incubated overnight at 4 °C with primary antibodies against VHL (Abmart, Shanghai, China, cat. No. PK76928S), HIF-1α (CST, Danvers, MA, USA, cat. No. 14179), HIF-2α (Boster, Wuhan, China, cat. No. A01248-1), VEGFA (Abclonal, Wuhan, China, cat. No. A0280), VEGFR2 (Boster, Wuhan, China, cat. No. A00901-3), and p-VEGFR2 (Affinity, Changzhou, China, cat. No. AF3279). The TBST-washed membranes were incubated with HRP-conjugated secondary antibody (ABclonal, Wuhan, China) for 1 h at room temperature, after which protein bands were developed with ECL and imaged using a Bio-Rad ChemiDoc XRS system.

### 4.20. Molecular Docking and Binding-Site Definition

The interaction mechanisms between the top 20 most abundant Kansui-DCM compounds and key HIF/VEGF pathway proteins were probed by molecular docking. The crystal structures of HIF-2α (PDB: 5TBM) and VEGFR2 (PDB: 4ASD) were obtained from the Protein Data Bank (PDB). AutoDock Vina v1.2.7 served to add polar hydrogens and Gasteiger charges to the receptors, after which the structures were stored as pdbqt files. For the small-molecule ligands, geometry optimization was performed using Gaussian 16W (Rev. C.01) software to obtain energetically stable three-dimensional conformations. Hydrogen atoms were explicitly added, and solvent effects were considered during the optimization process. The RESP (Restrained Electrostatic Potential) method was applied to compute and assign atomic partial charges, and AutoDock Vina was then used for docking simulations. Discovery Studio 2021 was employed to analyze the resulting protein–ligand interactions, while PyMOL v2.6 was used for visualization.

The docking search spaces were defined according to the binding regions of the co-crystallized ligands in the experimentally determined protein structures. For HIF-2α, the grid box was centered at X = 20.724 Å, Y = 3.744 Å, and Z = −13.308 Å. For VEGFR2, the grid box was centered at X = −28.606 Å, Y = 3.108 Å, and Z = −15.425 Å. The grid-box dimensions were set to 30 Å × 30 Å × 30 Å for both proteins, and the exhaustiveness parameter was set to 32.

The relatively large docking boxes were selected to cover the canonical ligand-binding cavities and their adjacent subpockets, thereby allowing the accommodation of compounds with different molecular sizes and conformational flexibility. Smaller compounds generally occupied binding regions overlapping with those of the co-crystallized ligands, whereas some larger compounds extended into neighboring subpockets because of their greater molecular dimensions. The docking scores are reported in kcal/mol in Table 2.

Formal redocking of the co-crystallized ligands was not performed in the present study. Therefore, the predicted poses and docking scores were used only for exploratory comparison and generation of structural hypotheses rather than as validated evidence of direct target binding or inhibition.

### 4.21. Molecular Dynamics

Molecular dynamics runs were conducted in GROMACS v2022.03. Parameterization employed AMBER99SB-ILDN for the protein and GAFF for the ligand, both prepared with AmberTools22. The solvated system (TIP3P water, 1.2 nm buffer) was neutralized by adding Na^+^. A cutoff of 1.0 nm was applied to short-range interactions, while long-range electrostatics were handled by the PME method. All bonds involving hydrogen were constrained via LINCS, permitting a 2 fs time step. After steepest descent energy minimization, the system was equilibrated for 100 ps in the NVT ensemble and 100 ps in the NPT ensemble, using the V-rescale thermostat and Berendsen barostat. Production simulations were conducted for 100 ns under NPT conditions at 300 K and 1 bar. The resulting trajectories were analyzed for RMSD, RMSF, hydrogen bond occupancy, radius of gyration, and solvent-accessible surface area.

### 4.22. Statistical Analysis

Statistical analysis was performed using SPSS Statistics (v.27.0, IBM, Armonk, NY, USA) and GraphPad Prism 8.0 (San Diego, CA, USA). All data are presented as mean ± SD from at least three independent experiments (in vitro) or from six biologically independent samples per group (in vivo). Normality was assessed using the Shapiro–Wilk test, and homogeneity of variances was evaluated using Levene’s test prior to parametric analysis. For comparisons between two groups, an unpaired two-tailed Student’s t-test was applied when data met parametric assumptions; otherwise, the Mann–Whitney U test was used. For comparisons involving three or more groups, one-way ANOVA was performed, followed by Dunnett’s test for comparisons against the control group. In cases where data violated the assumptions of normality or equal variances, the non-parametric Kruskal–Wallis test was used, followed by Dunn’s post hoc test. A *p*-value < 0.05 was considered statistically significant, and all tests were two-sided.

## 5. Conclusions

Collectively, these results integrated the traditional use of Kansui Radix with modern molecular pharmacology to investigate its anti-renal cell carcinoma (RCC) activity. For the first time, the dichloromethane extract of Kansui Radix (Kansui-DCM) was found to significantly impair RCC cell proliferation, migration, invasion, and angiogenesis in vitro and in vivo, while additionally reshaping the tumor immune milieu. Integrated transcriptomic, network pharmacological, and experimental validation approaches suggested that Kansui-DCM exerts its anti-RCC effects in association with suppression of the HIF/VEGF signaling pathway, potentially through a VHL-independent mechanism. However, causal demonstration of this mechanism requires further experimental validation. Furthermore, through computational docking and MD simulations, we uncovered two active compounds that bind stably to VEGFR2 and HIF-2α, thereby offering a molecular rationale for the observed pharmacological effects. These findings not only established a scientific rationale for the ethnopharmacological use of Kansui Radix in treating conditions resembling RCC but also laid a basis for translating it into a complementary treatment for RCC, pending further safety and efficacy evaluations.

## Figures and Tables

**Table 1:** The 20 chemical components with the highest concentrations in Kansui-DCM.

No.	Compounds	Formula	t_R_ (min)	Q1 (Da)	Q3 (Da)	Ionization Model	Level	Area
1	7-Methoxy-3-[1-(3-pyridyl)methylidene]-4-chromanone	C_16_H_13_NO_3_	1.9	268.10	136.06	[M + H]^+^	1	157,703,726
2	Miserotoxin	C_9_H_17_NO_8_	1.8	268.11	136.07	[M + H]^+^	3	152,725,571
3	4-(2,3,6-Trimethylphenyl)butan-2-ol	C_13_H_20_O	4.2	193.16	133.10	[M + H]^+^	2	123,644,986
4	Isoscopoletin	C_10_H_8_O_4_	4.1	193.05	178.03	[M + H]^+^	1	108,200,949
5	Scopoletin	C_10_H_8_O_4_	4.1	191.04	176.01	[M − H]^−^	1	81,493,196
6	2-Ethynyl-5-methoxybenzo-1,4-quinone	C_9_H_6_O_3_	5.8	163.04	77.04	[M + H]^+^	3	56,134,104
7	Isoferulic Acid	C_10_H_10_O_4_	4.0	193.05	134.04	[M − H]^−^	1	44,924,084
8	naphthisoxazol A	C_11_H_9_NO_2_	2.4	188.07	118.06	[M + H]^+^	1	42,955,140
9	3-Amino-2-naphthoic acid	C_11_H_9_NO_2_	2.3	188.07	118.06	[M + H]^+^	1	40,100,851
10	Methyl Orsellinate	C_9_H_10_O_4_	2.5	183.06	123.04	[M + H]^+^	2	38,370,129
11	Ferulate	C_10_H_10_O_4_	4.0	193.05	134.04	[M − H]^−^	1	37,122,407
12	4-Acetoxy-2-pyrrolidinone	C_6_H_9_NO_3_	2.1	144.07	84.04	[M + H]^+^	1	29,875,466
13	2,3-Dihydrosciadopitysin	C_33_H_26_O_10_	8.6	581.14	581.14	[M − H]^−^	3	24,278,245
14	Ligustrazine	C_8_H_12_N_2_	4.0	137.11	55.05	[M + H]^+^	1	23,371,665
15	Macrophypene B	C_18_H_28_O_2_	8.4	277.22	93.07	[M + H]^+^	3	21,831,290
16	Lycoperodine-1	C_12_H_12_N_2_O_2_	3.0	217.10	144.08	[M + H]^+^	3	21,112,453
17	2-Hydroxycinnamate	C_9_H_8_O_3_	4.1	163.04	119.05	[M − H]^−^	1	20,407,914
18	4-Hydroxy-3-methoxy-benzaldehyde	C_8_H_8_O_3_	4.0	151.04	136.02	[M − H]^−^	1	19,718,220
19	Kynurenine	C_10_H_12_N_2_O_3_	1.9	209.09	146.06	[M + H]^+^	2	19,593,358
20	Dihydrocaffeic acid	C_9_H_10_O_4_	2.9	183.07	165.06	[M + H]^+^	2	19,565,916

Geometric configuration was not confirmed due to the lack of authentic standards and NMR data; the reported name reflects the database entry only.

**Table 2:** Results of molecular docking.

Compound	Affinity (kcal/mol)	
	VEGFR2	HIF-2α
2,3-Dihydrosciadopitysin	−9.037	−7.201
2-Ethynyl-5-methoxybenzo-1,4-quinone	−6.139	−6.269
3-Amino-2-naphthoic acid	−7.735	−7.371
4-(2,3,6-Trimethylphenyl)butan-2-ol	−6.826	−6.536
4-Acetoxy-2-pyrrolidinone	−5.146	−5.203
4-Hydroxy-3-methoxy-benzaldehyde	−5.712	−5.715
7-Methoxy-3-[1-(3-pyridyl)methylidene]-4-chromanone	−8.426	−7.249
Dihydrocaffeic acid	−6.729	−6.357
Ferulate	−7.046	−6.407
Isoferulic Acid	−6.81	−6.432
Isoscopoletin	−6.823	−6.837
Ligustrazine	−4.997	−5.389
L-Kynurenine	−6.695	−6.894
Lycoperodine-1	−7.684	−7.563
Macrophypene B	−7.802	−5.562
Methyl Orsellinate	−8.031	−6.956
Miserotoxin	−5.75	−6.65
Naphthisoxazol A	−8.039	−6.987
Scopoletin	−6.84	−6.642
*trans*-2-Hydroxycinnamate	−7.221	−7.059

**Table 3:** Primers used for qRT-PCR.

Gene	Primer	Sequence (5′-3′)
*HIF-1α*	Forward primer	ACCTTCATCGGAAACTCCAAAG
	Reverse primer	CTGTTAGGCTGGGAAAAGTTAGG
*HIF-2α*	Forward primer	CTGAGGAAGGAGAAATCCCGT
	Reverse primer	TGTGTCCGAAGGAAGCTGATG
*VEGFA*	Forward primer	AGGGTCAAAAACGAAAGCGC
	Reverse primer	CGCGAGTCTGTGTTTTTGCA
*VEGFR2*	Forward primer	TTTGGCAAATACAACCCTTCAGA
	Reverse primer	GCAGAAGATACTGTCACCACC

## Data Availability

The data are available from the corresponding author upon reasonable request.

## Supplementary Materials

The following supporting information can be downloaded at https://www.mdpi.com/article/10.3390/ijms27167325/s1.

## Abbreviations

786-O cells	human renal clear cell adenocarcinoma cells
BCA analysis	bicinchoninic acid assay
BP	biological process
BSA	bovine serum albumin
CC	cellular component
CCK-8	cell counting kit-8
ccRCC	clear cell renal cell carcinoma
CD8	cluster of differentiation 8
CD31	platelet endothelial cell adhesion molecule-1
COX-2	cyclooxygenase-2
FBS	fetal bovine serum
FDR	false-discovery rate
FEL	free-energy landscape
GO	gene ontology
HIF	hypoxia-inducible factor
HIF-1α	hypoxia-inducible factor 1-alpha
HIF-2α	hypoxia-inducible factor 2-alpha
H&E	hematoxylin and eosin
IC_50_	half-maximal inhibitory concentration
IF	immunofluorescence
IHC	immunohistochemistry
Kansui-DCM	the dichloromethane fraction of Kansui Radix
KEGG	Kyoto Encyclopedia of Genes and Genomes
Ki67	Kiel 67
MF	molecular function
MM/PBSA	molecular mechanics/Poisson–Boltzmann surface area
PBS	phosphate-buffered saline
PVDF	polyvinylidene fluoride
PDB	Protein Data Bank
qRT-PCR	quantitative reverse-transcription polymerase chain reaction
RCC	renal cell carcinoma
RENCA cells	mouse renal carcinoma
RESP	restrained electrostatic potential
Rg	radius of gyration
RIPA	radioimmunoprecipitation assay
RMSD	root-mean-square deviation
RMSF	root-mean-square fluctuation
SASA	solvent-accessible surface area
SDS-PAGE	sodium dodecyl sulfate-polyacrylamide gel electrophoresis
TBST	Tris-buffered saline with Tween-20
UPLC-MS	ultra-high-performance liquid chromatography–mass spectrometry
VEGF	vascular endothelial growth factor
VEGFA	vascular endothelial growth factor A
VEGFR2	vascular endothelial growth factor receptor 2
VHL	von hippel–lindau tumor suppressor
WB	Western blot
WGCNA	weighted gene co-expression network analysis
