# Supplementary material for: Integrated WGCNA, Network Pharmacology, and UPLC-MS/MS Profiling for Investigating the Antitumor Effects of the Kansui Radix Dichloromethane Fraction Against Renal Cell Carcinoma with Experimental Validation
Source: Int J Mol Sci. 2026 Aug 16;27(16):7325. doi: 10.3390/ijms27167325 (PMC13513697; doi:10.3390/ijms27167325)
Supplement: Supplementary file 1 [file ijms-27-07325-s001.zip › ijms-4415238-supplementary.pdf]

Integrated WGCNA, network pharmacology, and UPLC-MS/MS profiling for investigating the antitumor effects of Kansui Radix dichloromethane fraction against renal cell carcinoma with experimental validation

Zhuoyang Cheng[a], Xinyue Chen[a], Shiqi Wang[a], Yunuan Bai[a], Jiangtao Zhou[a,b,c,*]

[a] *School of Pharmacy, Shanxi Medical University, Taiyuan 030001, China*

[b] *Medicinal Basic Research Innovation Center of Chronic Kidney Disease, Ministry of Education, Shanxi Medical University, Taiyuan 030001, China*

[c] *Shanxi Academy of Advanced Research and Innovation, 030032, Taiyuan, China*

[*]Corresponding author

E-mail address: zjt881206@sxmu.edu.cn

SI.1 The inhibition effects of Kansui on proliferation of cancer cells

After treating 786-O cells with different fractions of Kansui for 24 h, the CCK-8 assay revealed that the ethyl acetate and n-butanol fractions exhibited weaker inhibitory effects on RCC without dose-dependent relationships (Fig.S1A). In contrast, Kansui-DCM showed better inhibitory effect on 786-O cell proliferation in a dose-dependent manner, thereby identifying it as the active constituent responsible for the treatment of RCC.

Kansui-DCM was applied to 786-O and RENCA cells for durations of 24, 48, and 72 h, after which cell viability was evaluated. The Kansui-DCM demonstrated an inhibitory effect on the proliferation of both cell lines. The IC_{50} values (Fig.S1B) for Kansui-DCM against 786-O cells at 24, 48, and 72 h were determined to be 57.46, 45.59, and 36.38 μg/mL, respectively. In RENCA cells (Fig.S1B), the corresponding IC_{50} values were 38.37, 34.59, and 29.84 μg/mL. Based on these findings, 786-O cells were treated with Kansui-DCM at concentrations of 25, 50, and 75 μg/mL in subsequent assays, while RENCA cells received treatment at 10, 25, and 50 μg/mL.

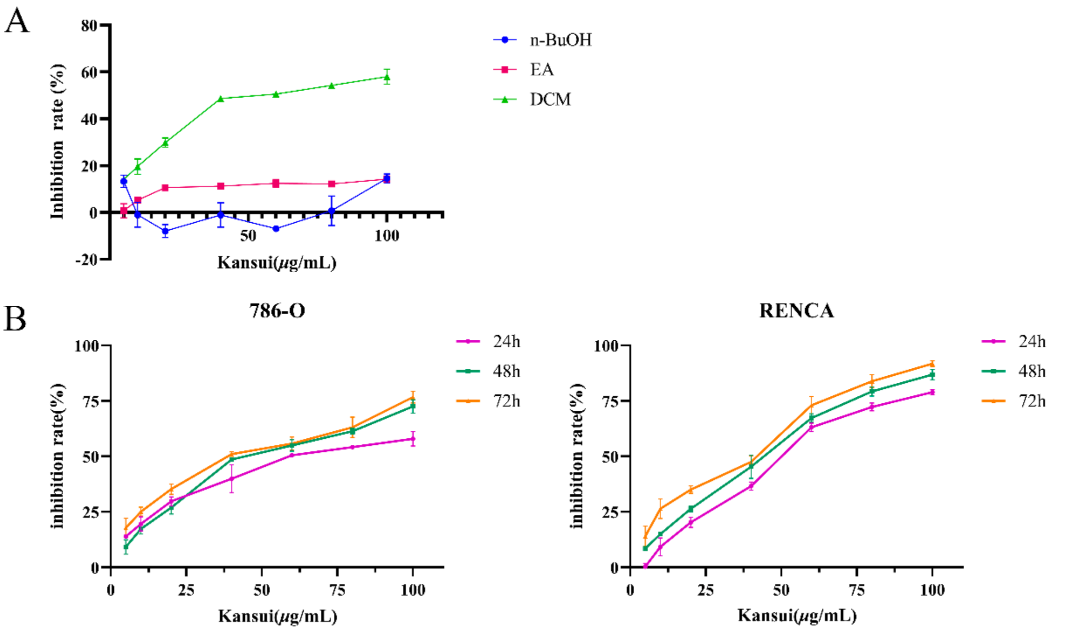

Fig.S1. The anti-proliferative effect of Kansui radix on renal cancer cells. A. Effects of different concentrations of Kansui radix from various parts on the proliferation of 786-O cells; B. Effect of Kansui-DCM on the proliferation of 786-O and RENCA cells.
